# Machine learning-based predictions of healthcare contacts following emergency hospitalisation using electronic health records

**DOI:** 10.1038/s41746-025-02138-4

**Published:** 2025-12-17

**Authors:** Konstantin Georgiev, Dimitrios Doudesis, Joanne McPeake, Nicholas L. Mills, Susan D. Shenkin, Jacques D. Fleuriot, Atul Anand

**Affiliations:** 1 Institute of Neuroscience and Cardiovascular Research, Queen’s Medical Research Institute, University of Edinburgh, Edinburgh, UK; 2 Department of Public Health and Primary Care, The Healthcare Improvement Studies Institute, University of Cambridge, Cambridge, UK; 3 Ageing and Health Research Group and Advanced Care Research Centre, Usher Institute, Edinburgh BioQuarter, University of Edinburgh, Edinburgh, UK; 4 Artificial Intelligence and its Applications Institute, School of Informatics, University of Edinburgh, Edinburgh, UK

**Keywords:** Diseases, Health care, Mathematics and computing, Medical research, Risk factors

## Abstract

Emergency care systems are challenged by the emergence of an ageing population, requiring tailored inputs facilitated by early care needs assessment. We examined the potential of Machine Learning algorithms to identify in-hospital healthcare contacts in older patients after emergency admission, developed from linked electronic health record (EHR) data within South-East Scotland. Gradient-boosting (XGBoost) prediction models were trained on frailty markers and nursing risk assessments to predict healthcare contacts, adverse outcomes and requirements for specialist input between arrival and 72 hours following admission. Across 98,242 patients, the predicted contact error rate varied between 49% at point of emergency attendance and 34% at 72 hours post-admission. Area-under-the-curve reached 0.89 in predicting need for urgent geriatric services, and 0.83 for in-hospital rehabilitation. Pressure ulcer risk and its documentation were predictive of received contacts. EHR data can predict granular estimates of in-hospital activity after ED attendance, facilitating quicker allocation to appropriate urgent care pathways.

## Introduction

Older patients with multiple long-term conditions, functional decline and geriatric syndromes comprise around two-fifths of emergency department (ED) attendances^1–4^. This complexity makes allocation of the optimum hospital pathway for admitted patients more difficult, increasing the risk of fragmented care or delays to appropriate specialist care. A recent study by Roussel et al. highlighted the negative implications of overnight stays in the ED, indicating a poorer likelihood of survival to discharge^5^. This emphasises the need for risk-aware triage, targeting patients who may be subject to serious functional decline. Current models in urgent care, necessarily focused on rapid throughput, often fail to differentiate risks between older patients within the critical 72-h window after admission^1,3,6^. A more rigorous understanding of the predictors of likely healthcare inputs needed within a hospital admission, including rehabilitation, could help reduce preventable adverse events, such as hospital-acquired disability^7,8^ or function decline^9^. In turn, risk-attuned systems could improve the allocation of limited multidisciplinary resources, such as physiotherapy time.

Modern electronic health record (EHR) systems now capture multidimensional indicators of frailty^10^, that could explain health and care contacts in hospital. Furthermore, every unique contact with a health provider can be measured and timestamped to reflect in-hospital activity^11^. These contacts data represent a novel endpoint to understand system use. We have previously shown how these data can describe differences in care patterns for patients experiencing early emergency readmission^12^, and explored rehabilitation delivery between patients with and without COVID-19 infection^13^.

Several studies have adopted machine learning (ML) approaches using routine data to predict hospital endpoints for patients attending the ED. Traditional outcomes, including conversion to inpatient admission^14^, length of stay^15^, in-hospital death^16^ or admission to critical care^17^ have been previously used to produce high-performing risk estimates. While these outcomes are robust and easily extractable from EHRs, such measures do not provide granular detail on the patterns or dose of care processes delivered. Further, outcomes like length of stay are often influenced by factors outside of healthcare control, such as a patient’s social needs or family support.

Our aim was to develop and evaluate ML models to forecast in-hospital healthcare contacts and key outcomes such as the requirement for rehabilitation or specialist care, using comprehensive but routinely available data from community and hospital EHRs.

## Results

### Cohort summary

We identified 208,477 eligible admissions following ED attendance across the three acute hospital sites. Our analysis cohort consisted of 98,242 unique patients (72 ± 12 years old, 51% women) who received a median of 7 [3, 19] nursing or rehabilitation contacts during their admission. Rehabilitation was provided for 40,946 (42%) patients, including contacts with physiotherapy (36,838, 37%), occupational therapy (22,489, 23%) and speech and language therapy (5839, 6%). Baseline characteristics by quintiles of total contacts are shown in Table 1. The majority of urgent hospitalisations were within RIE, followed by WGH, with around one-fifth of attendances derived from the lower-volume SJH.

**Table 1:** Patient characteristics at baseline grouped by in-hospital healthcare contact level

	All (*n* = 98242)	Healthcare contact frequency quintile				
		VL (*n* = 9687)	L (*n* = 26,994)	M (*n* = 21,841)	H (*n* = 19,981)	VH (*n* = 19,739)
Age (mean, SD)	72 (12)	68 (12)	69 (12)	71 (12)	73 (12)	71 (11)
Women	50,214 (51%)	4863 (50%)	13,246 (49%)	10,695 (49%)	10,423 (52%)	10,987 (56%)
SIMD in quintiles						
1 (most deprived)	15,735 (16%)	1634 (17%)	4415 (16%)	3633 (17%)	3101 (16%)	2952 (15%)
2–4	57608 (59%)	5665 (59%)	15818 (59%)	12852 (59%)	11842 (59%)	11431 (58%)
5 (least deprived)	24,899 (25%)	2388 (25%)	6761 (25%)	5356 (25%)	5038 (25%)	5356 (27%)
Attending hospital						
RIE	49,891 (50%)	5279 (55%)	14,290 (53%)	11,473 (53%)	9620 (48%)	9229 (47%)
WGH	29940 (31%)	2876 (30%)	7809 (29%)	6201 (28%)	6207 (31%)	6847 (35%)
SJH	18,411 (19%)	1532 (16%)	4895 (18%)	4167 (19%)	4154 (21%)	3663 (19%)
Medical condition history						
# Long-term conditions (median, IQR)	3 [2, 5]	3 [2, 5]	3 [2, 5]	3 [2, 5]	4 [2, 5]	4 [3, 5]
Simple Multimorbidity ($\ge$2 conditions)	33,534 (34%)	3440 (36%)	9656 (36%)	7535 (35%)	6546 (33%)	6357 (32%)
High-count Multimorbidity ($\ge$4 conditions)	48,655 (50%)	4107 (42%)	11,934 (44%)	10,603 (49%)	10,722 (54%)	11,289 (57%)
Physical-mental Multimorbidity ($\ge$1 physical and $\ge$1 mental condition)	36,096 (37%)	3547 (37%)	10,033 (37%)	8093 (37%)	7402 (37%)	7021 (36%)
Nursing assessments						
4AT score ($\ge$4, at risk)	6540 (7%)	132 (1%)	675 (3%)	1084 (5%)	1797 (9%)	2852 (14%)
MUST score ($\ge$2, at high risk)	5911 (6%)	119 (1%)	650 (2%)	842 (4%)	1377 (7%)	2923 (15%)
Waterlow score ($\ge$10, at risk)	17,023 (17%)	801 (8%)	2660 (10%)	3181 (15%)	4323 (22%)	6058 (31%)
Fall event within 6 months of admission	16,043 (16%)	350 (4%)	2149 (8%)	2721 (13%)	4052 (20%)	6771 (34%)
Walking dependence	17,074 (21%)	1412 (15%)	6341 (24%)	5434 (25%)	4338 (22%)	3105 (16%)
Bathing dependence	20,160 (21%)	1473 (15%)	6816 (25%)	5727 (26%)	4401 (22%)	2428 (12%)
Swallowing difficulties	1719 (2%)	26 (<1%)	136 (1%)	175 (1%)	333 (2%)	1049 (5%)
Secondary outcomes						
In-hospital death	6093 (6%)	202 (2%)	592 (2%)	931 (4%)	1720 (9%)	2648 (13%)
Extended stay ($\ge$14 days)	19,040 (19%)	307 (3%)	625 (2%)	671 (3%)	2113 (11%)	15,324 (78%)
Non-home discharge	12,938 (13%)	443 (5%)	1297 (5%)	1711 (8%)	2907 (15%)	5980 (30%)
Admission to Geriatric Medicine services	13,301 (14%)	392 (4%)	1044 (4%)	1445 (7%)	2843 (14%)	7577 (38%)

The contact frequency group was defined through binning the total health contacts variable into five equally-sized partitions. Values are displayed in patient counts (%) unless stated otherwise. Statistical testing: one-way analysis of variance (ANOVA) test in numerical data, Chi-squared test in categorical data. Contact frequency groups: VL—very low, L—low, M—medium, H—high, VH—very high.

At baseline, patients at higher contact quintiles were older, with a higher proportion of women. Distributions by quintiles of deprivation (Scottish Index of Multiple Deprivation (SIMD)) were similar between contact groups. There was a steady increase in the proportion of patients with over 4 long-term conditions recorded in their medical history over the contact frequency levels (57% in the VH group vs 41% in the VL group), but numbers with physical-mental multimorbidity were similar across all contact groups. Proportions of patients at risk of delirium (4AT), malnutrition (MUST) and pressure ulcers (Waterlow score), as well as prior fall events, also increased in line with increased healthcare contacts. Patients under specialist services had more consistent coding for ED metadata, and those under older adult and rehabilitation services had a higher proportion of complete nursing risk assessments (Supplementary Table 2).

Patients at the highest level of received contacts (VH) had the highest prevalence of in-hospital deaths (13%), admissions to older adult specialist services (38%) and non-home discharges (30%). The annual incidence rates for each secondary outcome varied (Supplementary Fig. 1). Older individuals with an outcome were more likely to be living in lower deprivation areas (Supplementary Fig. 2). The log-transformed distribution of contacts was significantly higher in patients who experienced each of the secondary outcomes (see Supplementary Fig. 3). A further summary of the baseline characteristics by each of these outcomes is available in Supplementary Tables 3–6.

### Health contact characteristics

Patterns of healthcare contact type are shown for each group in Table 2. Patients in the highest (VH) contact group had a median of 51 [34, 87] total hospital contacts, with a median 7 [5, 12] documented contacts each day of admission and significant multidisciplinary input (3 ± 1 unique provider types). This was characterised by an increase in both nursing (4 [3, 7] vs 2 [2, 3] contacts per day in H, *p* < 0.001), and rehabilitation contacts (93% vs 58% receiving rehabilitation in H, and 4 [2, 6] vs 2 [2, 3] contacts per day respectively, *p* < 0.001). Time to first rehabilitation contact was longer in the highest contact quintile (47 [21, 108] h in H vs 22 [15, 59] h in L), but the smaller proportion of patients within the lowest frequency (VL) group who received rehabilitation waited the longest to receive this (54 [21, 126] h). The total in-hospital healthcare contacts were elevated in all patients with any secondary outcome, with the highest level of care in those with extended stay and admission to older adult specialist services (see Supplementary Table 7). After log-transformation, the total number of contacts per individual remained significantly higher in RIE compared to WGH and SJH, particularly in rehabilitation provision (Supplementary Fig. 4, *p* < 0.001). The distributions of the average contacts per day, as well as the overall hospital length of stay, were different across the three sites, with WGH having the longest distribution tail for length of stay and RIE having the longest distribution tail for overall and rehabilitation contacts. Contact variability by admission seasons was present in rehabilitation provision, with significantly higher frequencies recorded during fall and winter time (Supplementary Fig. 5).

**Table 2:** Summary of the healthcare contacts distribution in the target population, grouped by in-hospital contact frequency

	All (*n* = 98,242)	Healthcare contact frequency quintile					*p*
		VL (*n* = 9,687)	L (*n* = 26,994)	M (*n* = 21,841)	H (*n* = 19,981)	VH (*n* = 19,739)	
Overall							
Healthcare contacts per admission^a^	7 [3, 19]	1 [1, 1]	3 [2, 4]	6 [5, 8]	14 [12, 18]	51 [34, 87]	<0.001
Healthcare contacts per admission day^a^	2 [2, 4]	1 [1, 1]	2 [1, 2]	2 [2, 3]	3 [2, 3]	7 [5, 12]	<0.001
Number of disciplines involved (mean, SD)^a^	2 (1)	1 (0)	1 (0)	1 (1)	2 (1)	3 (1)	<0.001
Nursing							
Nursing contacts per admission	5 [2, 12]	1 [1, 1]	3 [2, 3]	5 [5, 7]	11 [8, 14]	29 [18, 50]	<0.001
Nursing contacts per admission day	2 [1, 3]	1 [1, 1]	2 [1, 2]	2 [2, 2]	2 [2, 3]	4 [3, 7]	<0.001
Rehabilitation							
Received any rehabilitation (*n*, %)	40946 (42%)	1058 (11%)	3504 (13%)	6490 (30%)	11601 (58%)	18293 (93%)	<0.001
Rehabilitation contacts per admission^b^	6 [2, 17]	1 [1, 1]	2 [1, 2]	2 [2, 4]	5 [3, 8]	19 [10, 35]	<0.001
Rehabilitation contacts per admission day^b^	2 [2, 4]	1 [1, 1]	1 [1, 2]	2 [1, 2]	2 [2, 3]	4 [2, 6]	<0.001
Time to first rehabilitation contact (hours)^b^	42 [19, 90]	54 [21, 126]	22 [15, 59]	28 [17, 59]	45 [21, 90]	47 [21, 108]	<0.001

The contact frequency group was defined through binning the total health contacts variable into five equally-sized partitions. Values are displayed in patient counts (%) unless stated otherwise. Statistical testing: one-way analysis of variance (ANOVA) test in numerical data reported as mean (SD), Kruskal–Wallis *H* test in numerical data reported as median [IQR], Chi-squared test in categorical data. Contact frequency groups: VL—very low, L—low, M—medium, H—High, VH—very high.

^a^Includes nursing and rehabilitation disciplines.

^b^Rehabilitation data were calculated only for those who received at least one rehabilitation contact (defined as physiotherapy, occupational therapy or speech and language therapy).

### Model training summary

The preprocessed data for modelling included a stratified training set of 68,769 and a hold-out validation set of 29,743 samples at the point of ED attendance (median age 73 [62, 81], 51% women and 16% at the highest deprivation level in both sets). Using this model setup, the XGBoost regressor consistently outperformed other fine-tuned linear and non-linear estimators (see Supplementary Table 8). The training and validation sets were balanced by age, sex, SIMD, secondary outcome prevalence, total healthcare contacts, rehabilitation-specific contacts and number of healthcare disciplines involved during hospital stay (see Supplementary Table 9). The optimal model hyperparameters, identified using grid search, are listed in Supplementary Table 10.

### Model performance for in-hospital healthcare contact prediction

Performance summary in healthcare contacts prediction is shown in Table 3. The XGBoost regression model showed a clear reduction in error rate when forecasting in-hospital contacts as more hospital episode data were included. The conditional mean absolute percentage error (cMAPE) score indicated a slight to reasonable forecasting ability of the XGBoost regressor (error rate between 49% [48%–49%] and 34% [33%–35%]).

**Table 3:** Model performance summary for healthcare contacts prediction

Time point of prediction	Primary outcome metrics			
	MAE	cMAPE (%)^a^	BACC^b^	CKS^b^
ED Arrival	0.98 [0.98–0.99]	49% [49%–50%]	0.28 [0.28–0.29]	0.34 [0.33–0.35]
Hospital admission	0.93 [0.93–0.94]	47% [46%–47%]	0.32 [0.31–0.33]	0.43 [0.42–0.44]
24 h post-admission	0.86 [0.85–0.87]	42% [41%–43%]	0.32 [0.31–0.32]	0.46 [0.45–0.47]
48 h post-admission	0.82 [0.81–0.83]	36% [35%–37%]	0.32 [0.32–0.33]	0.46 [0.45–0.48]
72 h post-admission	0.80 [0.79–0.81]	34% [33%–34%]	0.32 [0.32–0.33]	0.46 [0.45–0.47]

Values are reported with 95% confidence intervals. Metrics: MAE—mean absolute error, cMAPE—conditional Mean Absolute Percentage Error, BACC—balanced accuracy score, CKS—Cohen’s Kappa score. GMS—geriatric medicine services.

^a^Estimated by masking 0 values (no linked contacts), due to inherent limitations of MAPE.

^b^Metrics estimated through quintile-based discretisation of the predicted healthcare contacts.

**Fig. 1:**
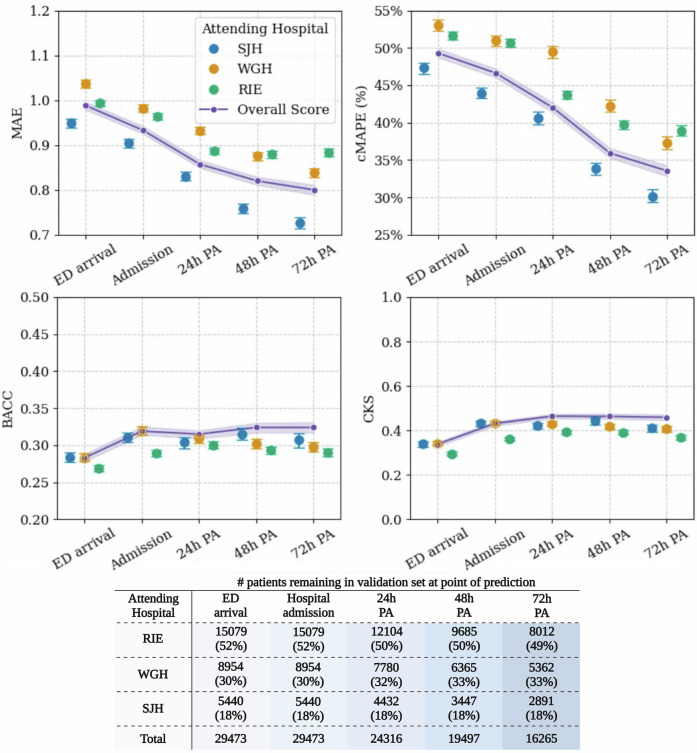
Leave-one-hospital-out validation for in-hospital contacts forecasting across prediction timepoints. Balanced accuracy and Cohen’s Kappa score were estimated after quintile-based discretisation of predictions into five frequency levels: ‘Very Low’, ‘Low’, ‘Medium’, ‘High’ and ‘Very High’. MAE mean absolute error, cMAPE conditional mean absolute percentage error, estimated by masking 0 values from the estimation.

The leave-one-hospital-out validation revealed a generalisation gap between the mean absolute error (MAE) and cMAPE scores across hospital sites (Fig. 1). When validated on the smaller sample of SJH patients, the MAE reduced up to 23%, and the cMAPE reduced by 19%, substantially lower than that of the WGH and RIE samples. Compared to the original performance, the cMAPE was 37% [36%-38%] for the WGH sample and 38% [38%-39%] for the RIE sample. The BACC and Cohen’s Kappa scores (CKSs) were more similar but slightly lower than the baseline at 72 h post-admission. By this time, 60% of patients in the validation set within the WGH sample remained in the study, while 53% remained within both the RIE and SJH groups.

Considering the ED arrival model as the baseline, we achieved an overall 5% reduction in error at point of hospitalisation (MAE = 0.93 [0.93–0.94] from 0.97 [0.97–0.98]), 12% reduction at the first day of admission (MAE = 0.86 [0.85–0.87]) and 18% reduction by the third day of admission (MAE = 0.80 [0.79–0.81]). Use of other linear and non-linear regression estimators showed an increase in error rate, compared to XGBoost (Supplementary Table 8). The 10-fold cross-validation strategy revealed comparable MAE and cMAPE measures for the baseline model (Supplementary Table 11).

The BACC score indicated more limited classification ability in estimating the contact frequency category across five intensity groups (between 0.28 [0.28–0.29] and 0.32 [0.32–0.33], with random choice being 0.20). The quality of stratification based on measures of agreement could be treated as fair (0.34 [0.33–0.35] at the point of ED attendance) up to moderate (0.46 [0.45–0.47] by the third day of admission). Additional error analysis on the validation set (Supplementary Fig. 6) revealed that the algorithm had the highest success rate when classifying patients requiring the highest level of contacts (37–46% across prediction timepoints). Meanwhile, classification of medium levels of care was most challenging (22–24%). A stratified analysis by age groups revealed significantly higher forecasting quality among the younger groups at point of ED attendance (MAE = 1.1 in 90+ group vs 0.87 in 50–59 group), but this gap in quality reduced in the later prediction windows (Supplementary Fig. 7). These differences were not observed across SIMD groups (Supplementary Fig. 8). After adjusting for non-home discharge outcomes (Supplementary Fig. 9), patients at the highest level of received contacts had the lowest probability of death by day 10 after ED arrival (1% vs 7% in medium-high). However, the survival advantage of the highest contact group diminished by day 30.

After testing the model on survivors to discharge (*n* = 92,149), we observed similar performance trajectories across the four regression quality metrics (Supplementary Fig. 10). However, when validated on patients strictly admitted within the COVID-19 lockdown period, performance improved substantially with the cMAPE score dropping to 24% and the MAE reaching 0.68 after 72 h of admission (Supplementary Fig. 11).

### Model performance for secondary hospital outcomes

**Fig. 2:**
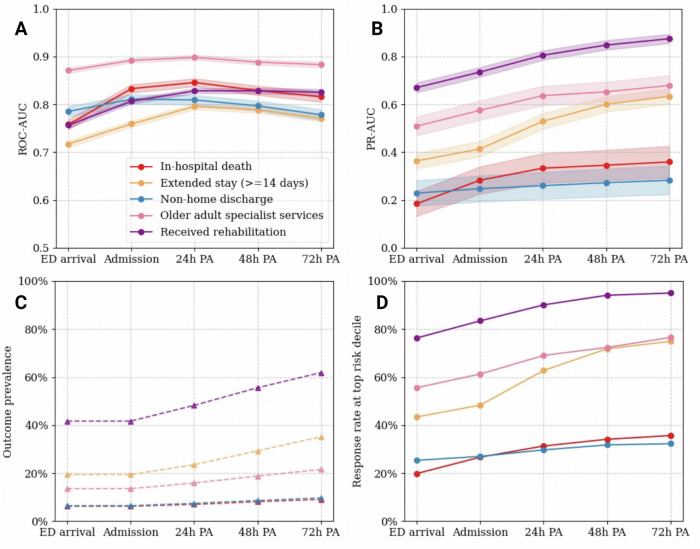
Performance trajectory curves for the binary hospital outcomes reported with 95% confidence intervals across the five prediction timepoints. **A** Receiver operating characteristic area under the curve. **B** Precision-recall area under the curve indicated by the straight coloured lines, along with the change in outcome prevalence due to patient exclusion over time, marked by dashed lines and triangle points. **C** Proportion of patients with the health outcome over time. **D** Patient response rate after risk stratification, measuring the % of correctly captured individuals at the 10th decile of risk with the respective hospital outcome.

The XGBoost classification models achieved moderate to robust performance on the hospital endpoints, considering the same input features as the health contact forecasting model. Figure 2A, B shows the change in the trajectory of the receiver operating characteristic area-under-the-curve (ROC-AUC) and precision-recall area-under-the-curve (PR-AUC) scores, detailing overall discrimination ability and positive event classification rate, respectively. Predictive quality at 24 h from admission was excellent in admissions to older adult specialist services (ROC-AUC = 0.90 [0.89–0.91], PR-AUC = 0.64 [0.60–0.68]). At this stage of prediction, discrimination was also efficient for the forecasting of any rehabilitation requirements (ROC-AUC = 0.83 [0.82–0.83], PR-AUC = 0.81 [0.79–0.83]).

Across all prediction timepoints, there was a notable increase in discrimination ability for in-hospital death (by 9%, 0.85 [0.84–0.85] from 0.76 [0.75–0.77]), extended stay (by 8%, 0.80 [0.79–0.80] from 0.72 [0.71–0.72]) and any rehabilitation delivery (by 7%, 0.83 [0.82, 0.83] from 0.76 [0.75–0.76]) within the first day of admission. By comparison, the positive class detection rate (PR-AUC) increased steadily over time. The tradeoff between precision and recall was reasonable in the presence of imbalanced data, as the random choice threshold for adverse event detection (equal to the incidence rate of the target time point) was two to three times lower than the reported scores.

Table 4 showed that model sensitivity was maximised either at point of hospitalisation (0.75 [0.73, 0.77]) for non-home discharge or within 24 h of admission (0.81 [0.79–0.83] for in-hospital death, 0.76 [0.75–0.77] for extended stay, 0.78 [0.78–0.79] for rehabilitation and 0.86 [0.85–0.87] for admission to geriatric services), indicating that a majority of cases could be flagged early in the admission. Selection of individuals requiring rehabilitation was close to ideal (Fig. 2D), ranging from a 79% response rate at ED arrival up to 95% by the third day of admission.

**Table 4:** Model performance summary on the binary hospital outcomes across prediction timepoints

Time point of prediction	Secondary outcome metrics					
	ROC-AUC	PR-AUC	Sensitivity	Specificity	PPV	NPV
ED Arrival						
In-hospital death	0.76 [0.75–0.77]	0.18 [0.13–0.24]	0.74 [0.72–0.76]	0.64 [0.64–0.65]	0.12 [0.12–0.13]	0.97 [0.97–0.98]
Extended stay	0.72 [0.71–0.72]	0.36 [0.33–0.39]	0.72 [0.71–0.73]	0.61 [0.61–0.62]	0.31 [0.30–0.32]	0.90 [0.90–0.91]
Non-home discharge	0.79 [0.77–0.80]	0.23 [0.18–0.28]	0.68 [0.66–0.70]	0.77 [0.76–0.77]	0.17 [0.16–0.18]	0.97 [0.97–0.97]
Admission to GMS	0.87 [0.87–0.88]	0.51 [0.47–0.55]	0.81 [0.80–0.82]	0.77 [0.77–0.78]	0.36 [0.35–0.37]	0.96 [0.96–0.97]
Received rehabilitation	0.76 [0.75–0.76]	0.67 [0.65–0.69]	0.70 [0.70–0.71]	0.69 [0.68–0.70]	0.62 [0.61–0.63]	0.77 [0.76–0.77]
Hospital admission						
In-hospital death	0.83 [0.82–0.84]	0.28 [0.23–0.34]	0.79 [0.77–0.81]	0.72 [0.71–0.72]	0.16 [0.15–0.17]	0.98 [0.98–0.98]
Extended stay	0.76 [0.75–0.77]	0.41 [0.38–0.44]	0.72 [0.70–0.73]	0.68 [0.67–0.69]	0.35 [0.34–0.36]	0.91 [0.90–0.91]
Non-home discharge	0.81 [0.80–0.82]	0.25 [0.19–0.30]	0.75 [0.73–0.77]	0.73 [0.73–0.74]	0.16 [0.16–0.17]	0.98 [0.98–0.98]
Admission to GMS	0.89 [0.89–0.90]	0.58 [0.54–0.61]	0.82 [0.81–0.83]	0.81 [0.80–0.81]	0.40 [0.39–0.41]	0.97 [0.96–0.99]
Received rehabilitation	0.81 [0.80–0.81]	0.74 [0.72–0.76]	0.75 [0.74–0.76]	0.72 [0.72–0.73]	0.66 [0.65–0.67]	0.80 [0.80–0.81]
24 h post-admission						
In-hospital death	0.85 [0.84–0.85]	0.33 [0.27–0.40]	0.81 [0.79–0.83]	0.72 [0.71–0.73]	0.18 [0.17–0.19]	0.98 [0.98–0.98]
Extended stay	0.80 [0.79–0.80]	0.53 [0.50–0.56]	0.76 [0.75–0.77]	0.69 [0.68–0.70]	0.43 [0.42–0.44]	0.90 [0.90–0.91]
Non-home discharge	0.81 [0.80–0.82]	0.26 [0.20–0.32]	0.71 [0.69–0.73]	0.77 [0.76–0.77]	0.20 [0.19–0.21]	0.97 [0.97–0.97]
Admission to GMS	0.90 [0.89–0.90]	0.64 [0.60–0.68]	0.86 [0.85–0.87]	0.78 [0.77–0.78]	0.42 [0.41–0.43]	0.97 [0.96–0.97]
Received rehabilitation	0.83 [0.82–0.83]	0.81 [0.79–0.83]	0.78 [0.78–0.79]	0.72 [0.71–0.73]	0.72 [0.72–0.73]	0.78 [0.78–0.79]
48 h post-admission						
In-hospital death	0.83 [0.82–0.84]	0.35 [0.28–0.41]	0.72 [0.69–0.74]	0.78 [0.77–0.78]	0.22 [0.21–0.23]	0.97 [0.97–0.97]
Extended stay	0.79 [0.78–0.80]	0.60 [0.57–0.63]	0.74 [0.73–0.75]	0.69 [0.68–0.70]	0.50 [0.49–0.51]	0.86 [0.86–0.87]
Non-home discharge	0.80 [0.79–0.81]	0.27 [0.21–0.33]	0.75 [0.73–0.77]	0.71 [0.70–0.71]	0.19 [0.18–0.20]	0.97 [0.97–0.97]
Admission to GMS	0.88 [0.88–0.89]	0.65 [0.61–0.69]	0.84 [0.82–0.85]	0.78 [0.78–0.79]	0.47 [0.46–0.48]	0.95 [0.95–0.96]
Received rehabilitation	0.83 [0.82–0.83]	0.85 [0.83–0.87]	0.74 [0.73–0.75]	0.77 [0.76–0.78]	0.80 [0.79–0.81]	0.70 [0.69–0.71]
72 h post-admission						
In-hospital death	0.82 [0.81–0.83]	0.36 [0.29–0.42]	0.75 [0.73–0.78]	0.74 [0.73–0.74]	0.22 [0.21–0.23]	0.97 [0.96–0.97]
Extended stay	0.77 [0.76–0.78]	0.63 [0.60–0.66]	0.74 [0.73–0.75]	0.66 [0.65–0.67]	0.54 [0.53–0.55]	0.83 [0.82–0.83]
Non-home discharge	0.78 [0.77–0.79]	0.28 [0.22–0.34]	0.70 [0.68–0.72]	0.72 [0.71–0.72]	0.21 [0.20–0.22]	0.96 [0.95–0.96]
Admission to GMS	0.88 [0.88–0.89]	0.68 [0.64–0.72]	0.81 [0.80–0.83]	0.80 [0.79–0.80]	0.52 [0.51–0.53]	0.94 [0.93–0.94]
Received rehabilitation	0.83 [0.82–0.83]	0.87 [0.86–0.89]	0.77 [0.76–0.78]	0.73 [0.72–0.74]	0.82 [0.81–0.83]	0.66 [0.65–0.67]

Values are reported with 95% confidence intervals. Metrics: BACC—balanced accuracy score, CKS—Cohen’s Kappa score, GMS—geriatric medicine services.

### Feature importances for healthcare contacts prediction

**Fig. 3:**
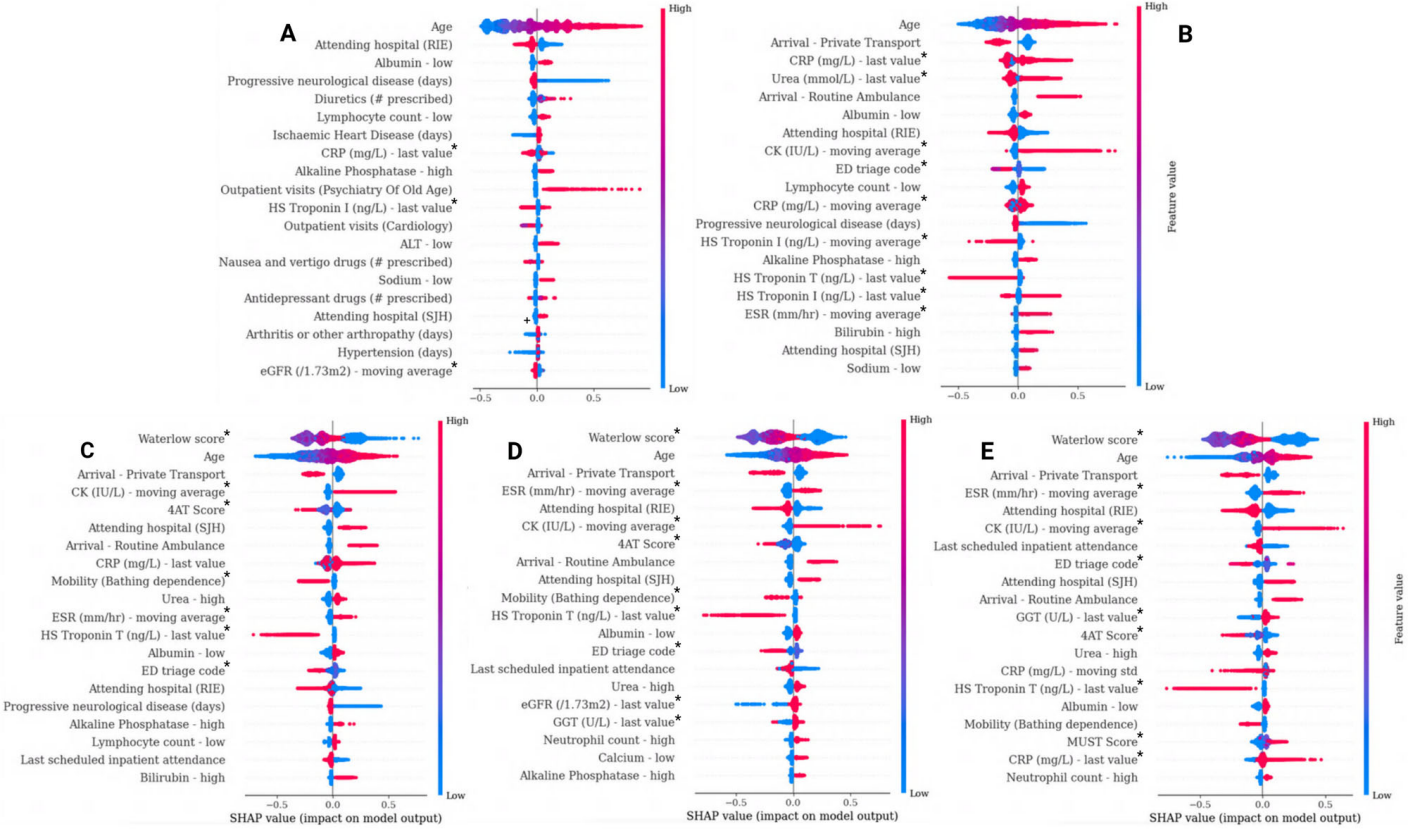
TreeSHAP feature importance summaries for the forecasting of in-hospital healthcare contacts across prediction timepoints. Density plots include the top 20 ranked predictors (beginning with the most important, independent of risk direction). Low values (blue) indicate individual association with less than average healthcare contacts, while high values (red) indicate association with above average contacts. **A** Point of ED arrival, **B** Point of hospital admission, **C** 24 h post-admission, **D** 48 h post-admission, **E** 72 h post-admission. Blood markers: CRP C-reactive protein, ALT alanine transaminase, CK creatine kinase, ESR erythrocyte sedimentation rate, eGFR estimated glomerular filtration rate, GGT gamma-glutamyl transferase. *Marked features include missing data indicated as −1 (lowest value: light blue).

The TreeSHAP density plot highlighted the top predictors of received healthcare contacts and the direction of their relationship (Fig. 3). Most of the top predictors were linked to older age, ED measurements, nursing assessments, blood testing and morbidities.

Interestingly, after the first day of admission, the Waterlow score became the most prominent indicator of received healthcare contacts, surpassing age in importance. Patients at moderate to high risk of pressure ulcers were predictive of less than average contacts, while no documented assessment was predictive of receiving more contacts. A similar pattern was also present in the 4AT score and mobility score specific to bathing dependence. In Supplementary Fig. 12, we identified possible relationships with survivorship bias within these variables, as patients who died in hospital and were assessed to be at no or low documented risk for delirium and pressure ulcers still had significantly more nursing and rehabilitation contacts compared to survivors at lower risk. However, the distributions were similar in the higher risk ranges. Patients with no documented nursing risk assessments were likely to receive more contacts, possibly as a result of complications in care continuity (Supplementary Fig. 13). The frequency of rehabilitation contacts was notably much higher in patients with no documented Waterlow scores.

In contrast, recent diagnoses for progressive neurological disease (including dementia) were predictive of higher contact counts. Mode of arrival is a known predictor of disease severity and health outcomes. Here, it was apparent that private transport was linked to fewer healthcare inputs in the hospital, while arrival via ambulance indicated higher contacts. Attendance to RIE was likely to influence lower overall contacts, while attendance to SJH was likely to influence higher than average contacts, particularly when staying in hospital for more than 48 h. Among the lab tests, C-reactive protein (CRP), Urea and Creatine Kinase (CK) were the most prominent predictors. In this case, elevated CK or CRP (as a result of tissue damage or inflammation), or elevated urea levels (as often seen in dehydration) were associated with having more healthcare contacts.

## Discussion

We have described the clinical utility of an ML approach for predicting in-hospital healthcare contacts for patients from the point of ED attendance, developed entirely using routinely entered data. Forecasting for future healthcare contacts was feasible but had limited precision at the point of ED arrival, reaching moderate performance within 72 h of admission. Model agreement across care contact levels was limited, but certainty was greatest for those receiving the most care contacts. There was a notable generalisation gap across hospital sites, as the error rate was lower across timepoints in SJH, compared to RIE and WGH. The secondary models had excellent discrimination ability for in-hospital death and requirement for older adult specialist and rehabilitation services. Within 24 h of admission, 9 out of 10 individuals within the top decile of risk for requiring rehabilitation were correctly captured. We developed models for predicting flexible endpoints of in-hospital activity, covering relevant ageing, frailty and multimorbidity predictors captured during and before hospital admission. This identified the potentially hidden predictive utility of routine nursing risk assessments and blood tests measured at the critical points of patient movement through hospital and emergency services. Our approach demonstrates the potential of healthcare contacts forecasting while acknowledging the current limitations in routine healthcare data for care pathway optimisation, with the future aim to move from queue-based resource allocation to person-centred, data-driven approaches and reduce delays to accessing specialist care.

Age, mode of ED arrival, nursing assessments (pressure damage and delirium risk) and laboratory tests (infection, tissue damage and dehydration) were among the top indicators of received healthcare contacts. This reflects a combination of chronic determinants of received healthcare and potential surrogates of acute illness. The Waterlow skin health score was the most prominent predictor for fewer contacts after 24 h of admission. A high risk of pressure ulceration often indicates underlying frailty and a limited functional baseline prior to the acute illness precipitating hospitalisation. While increasing nursing attention is clearly indicated, there may be limited scope for rehabilitation contacts in this population, as there may be no goal to regain or improve mobility. Delirium, a form of acute cognitive decline, was also associated with reduced contacts. This contrasted with dementia, where higher interactions were documented. This distinction between acute and chronic cognitive impairment is clinically meaningful, with patients suffering from delirium often unable to participate in rehabilitation until the acute driver for this condition settles. There may also be some competing risk here, given the high association between delirium and mortality, effectively reducing observed inpatient contacts in this group^18^.

Importantly, the lack of documentation for pressure ulcer, delirium and mobility assessments was also predictive of more received contacts later in the pathway. This may be a signal of gaps in care coordination, increasing the likelihood of triggering further clinical reviews. For delirium, it is well-known that underreporting and underdiagnosis in hospital practice can lead to high rates of complications and subsequent adverse events^19^. Missing mobility or skin integrity assessments can also delay necessary repositioning interventions^20^, including pain control and wound management, directly contributing to unmet physical needs.

In our secondary analyses, we compared the quality of prediction in binary hospital endpoints, including in-hospital death, extended stay, non-home discharge, admission under older adult specialty services and requirement for rehabilitation. Discrimination ability was excellent for the requirements of older adult specialist services, even at the point of hospitalisation, suggesting a potential clinically important role in early triage of these patients to appropriate specialist care. Delivering such individualised care is often limited by system constraints^21^, despite numerous trials showing benefits of these person-centred approaches^22,23^. Some of the results from this observational study point to the future utility of data-driven approaches to support proactive care in high-risk patients. For example, our models show excellent discrimination for death during the hospital admission, using data obtained within the first 24 h of attendance. This could support clinicians in decisions on placement for appropriate monitoring and continuity of care where the risk of deterioration may not be obviously apparent, or to allow sensitive and targeted conversations to prepare patients and families. The tradeoff between precision and recall suggested a reasonable classification rate across all hospital endpoints, but generally better sensitivity than specificity. This is common in screening applications to support clinical teams, with an acceptance of more false negative cases to maximise identification of those at the highest risk of the harmful outcome.

A few ML studies have employed adjacent approaches to capture healthcare utilisation. Our study uses logged care contacts during hospitalisation with significant completeness across the full population. Ocagli et al. used a two-step approach using preliminary clustering of hospitalised individuals based on the Barthel index^24^, before classifying individuals into high- and low-consumption categories^25^. Tello et al. provided an ML approach for predicting healthcare utilisation via in-hospital bed days, which aligns with our key objective of proposing this work to help design optimum pathways in older individuals^26^. We argue that predicting received healthcare contacts can yield considerable benefits for both triage and service planning, reflective of requirements for staffing and multidisciplinary care.

Definitions of healthcare utilisation in hospitals vary significantly in the literature. For example, Blecker et al. use the term ‘EHR interactions’ to provide comparisons for delivered hospital care, specifically focusing on variation between workload on weekdays and weekends^27,28^. The study highlights the inherent fluctuations in healthcare delivery that vary by the day of admission. This variation in available resourcing makes individualised targeting to patients at higher contact levels even more important. A number of other works used claims data linked to continuity of care measures to study all-cause or disease-specific readmission risk^29–31^. Length of stay is another health outcome that is often used to determine the complexity of treatment. However, this often does not reflect received healthcare input, as discharge delays could occur while waiting for the availability of community and social care^32^.

Our study has several important strengths. We provide an extensive ML framework developed from rich primary and secondary care data across multiple hospitals. We used a reproducible and interpretable data pipeline to extract, select and explain features across a range of data sources from the EHR. Our derived nursing risk assessments provide a novel set of data features that do not form part of usual disease-based coded datasets, but which capture important markers of frailty early in a hospital admission. Our use of routine nursing and rehabilitation contacts provides a novel endpoint to explain healthcare utilisation. While there are definite relationships between this and administrative measures like length of stay, the prediction of individual elements of care could be pivotal for targeting resources and staff allocation. We have previously shown the added value of healthcare contacts in relation to early readmission risk after hospital discharge, with this association adding information beyond length of stay^12^. This approach is scalable and transferable to other health services using EHR systems, as we use a simple contact measure that should be extractable from most EHRs as auditable data. Our models generalised better when independently validated in attendance during the COVID-19 lockdown period in the UK. This may be indicative of the predictive qualities of the routinely collected data and their robustness to rapid shifts in healthcare delivery. In a previous study, we evaluated care coordination during the pandemic within NHS Lothian services, identifying substantial shifts in the variation of care in the acute hospitalisations of older adults^29^. Although patient care pathways did not appear to be streamlined in patients with COVID-19 infection, it is possible that the documentation of care became more consistent during this period due to reduced routine activities and a focus on respiratory interventions and infection control.

We must also acknowledge some important limitations. Our XGBoost regressor produces an important baseline for predicting future care contacts but falls short, particularly at the early stages of ED arrival, where forecasting accuracy is weaker. While this does improve at later prediction timepoints, performance would suggest that future deployment would require cautious use with clinical oversight, as is appropriate for any decision support tool. We must also accept that the error rate was poorer when independently validated against more acute and complex presentations, typically present in the higher-volume RIE and WGH sites. This is not particularly surprising as RIE and WGH act as regional referral centres for major trauma, where many patients arrive with acute or life-threatening conditions that require rapid escalation. As a district general hospital, SJH still offers services for mental health and skin injuries, but does not typically support major trauma cases, which lowers the comparative level of patient acuity at presentation. Thus, post-hoc calibration may be needed to correct for systematic differences in prediction between tertiary and district-level hospitals with regard to the serving of specialist care.

As with all models developed on retrospective data, it is important to acknowledge that predicted outcomes reflect current practice rather than optimised care. Application must not increase inequalities by bluntly refocusing activity based entirely on model predictions, as this may embed ineffective care. We also recognise that clinical application of such tools would require significant improvements in real-time data linkage of primary and secondary healthcare data to make these model outputs visible to hospital clinicians at the point of patient attendance. Initiatives such as NHS England’s Federated Data Platform^33^ are likely to move closer to this ambition, but these will require sustained investment to support appropriate use of ML/AI models to improve patient care.

Further inclusion of health and frailty markers prior to hospitalisation, such as the electronic Frailty Index^10^ from primary care, or Hospital Frailty Risk Score^34^ and more granular data on illness acuity, such as vital signs that comprise the National Early Warning Score (NEWS2)^35^, could significantly improve generalisation. We have used blood results from ED as a surrogate of illness acuity, but these might be less reliable markers in older adults^36^. Our significant findings on the Waterlow score might suggest that this is capturing severe illness acuity better than other variables. Further observational research integrating these data and engagement with stakeholders could aid in determining a suitable error threshold and useful timepoints for model outputs to inform resource allocation. We must acknowledge the potential implications of survivorship bias in a retrospective study cohort. Documentation in routine screenings, such as delirium and pressure ulcer risk, may cause overrepresentation of low-risk patients with future in-hospital death, because of unanticipated disease progression precluding comprehensive assessment. Subsequently, these patients may be less likely to have documented nursing and rehabilitation assessments, resulting in more flawed predictions. Thus, a multi-state or multi-label modelling approach accounting for the competing risk of death when estimating future contacts may prove to be more effective in strengthening clinical validity. We also recognise that our current definition of a healthcare contact does not provide a holistic representation of the care delivered across the hospital service. Patients are likely to receive inputs from many other members of a multidisciplinary team, such as doctors, pharmacists and dieticians, amongst many others, which could not be included in our models. Our predictive modelling reflects the downstream risk associated with conditions, lab testing and prescribing recorded in primary care, but does not explicitly capture GP interventions (e.g. preventative management, early referrals) that may influence acuity at hospital presentation. Future research could enrich models by incorporating data on GP consultations, care access patterns, and prescribing trajectories, which may provide additional insight into how primary care influences hospital demand. Importantly, our contacts measure captures EHR interaction in clinical notation, but this does not reflect every nursing or rehabilitation interaction with a patient.

This foundational work proposes the use of ML algorithms to improve hospital resource allocation using routinely entered healthcare data. We demonstrate that ML algorithms can use routinely entered healthcare data to predict fine-grained elements of care pertaining to resource allocation in urgent care. Data-driven methods are needed to improve the efficiency and effectiveness of care pathways in older people. We have demonstrated methods that could promote equitable, targeted and person-centred care, which is of great importance in complex systems where capacity and organisation are critical constraints.

## Methods

### Study design and participants

To determine our study population, we used retrospective research-linked EHR data in adults aged ≥50 years across three acute hospitals in the Lothian region of South-East Scotland:

- Royal Infirmary of Edinburgh (RIE), Edinburgh: a tertiary hospital handling major trauma and a wide range of specialist acute care
- Western General Hospital (WGH), Edinburgh: a tertiary hospital and the regional centre for cancer treatment and specialist neurology
- Saint John’s Hospital (SJH), Livingston: a general hospital supporting district-level emergency services with a focus on elective care

Data linkage extracted eligible hospitalisations between 1st April 2016 and 1st February 2024. Hospital admission episodes were included if linked to prior ED attendance. We included only consecutive non-surgical admissions, excluding episodes with no transfer of care after ED arrival, immediate admissions under surgical services or very short admissions without any linked nursing or rehabilitation contact. We used the first (index) admission meeting the above criteria per patient to generate an analysis cohort of unique individuals.

### Healthcare contacts data for outcome derivation

To derive the endpoints for received in-hospital contacts, we used available entries from nursing staff and specialised rehabilitation providers in physiotherapy, occupational therapy and speech and language therapy. Our primary dataset used hospital care contact data, collated from a shared EHR system (TrakCare, InterSystems, MA) used in the region. This includes each record of a delivered patient interaction where a clinical note was entered in a patient record. All three hospitals included in the study used fully digital records for recording clinical care throughout this period. Timestamped clinical notes were classified by the specialty of the healthcare professional. Contacts from doctors and other clinicians, such as pharmacists, were not available for this study. We linked these data to eligible episodes between index hospital admission and discharge to calculate the total number of health contacts delivered to each patient.

### Other linked data sources

Relevant hospital episodes were determined using the Scottish Morbidity Record (SMR) 01^37^, a national administrative dataset provided by Public Health Scotland that was linked to the hospital EHR system, to add additional information about the associated ED attendance (triage urgency codes and mode of arrival). We used the attending hospital code and season of admission as additional baseline features. Inpatient discharge codes were used to determine whether episodes led to home discharges or transfers to a non-home environment, including continued complex care. Similarly, previous attendances in outpatient clinics were linked and summarised by specialty using the SMR00 dataset. Linked demographics data included age, sex and the SIMD^38^, measured in quintiles and describing relative socioeconomic deprivation by postcode area within the region.

Medication history was obtained from the Scottish Prescribing Information System, containing records of dispensed community prescriptions at any point prior to the index admission. Prescriptions were categorised by Anatomical Therapeutic Chemical (ATC) coding into 14 common prescribing groups. Laboratory data for common haematology and biochemistry tests were included from both community and hospital records, including the index episode, extracted from the local EHR system (Supplementary Table 1).

Morbidities were extracted using GP Read codes from primary care (Read version 2 data) and ICD-10 coding from secondary care data (SMR01). These were consolidated into long-term condition groups based on code lists produced by the CALIBER research group^39^, published in the Health Data Research UK phenotyping library. We utilised 25 high-level condition groups developed from a previous observational cohort study as training inputs^12^. Patient deaths were determined via linkage with the National Records of Scotland registration data.

EHR specialty codes were used to determine which patients were managed by older adult specialist (geriatric medicine) services. Finally, data from a range of nursing risk assessments were linked from the EHR, using a bundle of measures mandated within the first 24 h of all older adult hospital admissions as part of accreditation and care assurance standards^40^. These included assessments for mobility, falls, nutrition, bowel and bladder function, infection prevention, rationale for bedrail use, and three routinely coded risk scores:

- The 4 As test (4AT)^41^ score: a cumulative score between 0 and 12, where a score $\ge$4 indicates possible delirium.
- The Waterlow score^42^: a cumulative score between 0 and 25, used to estimate risk of developing pressure ulcers (bedsores), where a score $\ge$10 indicates the presence of risk and recommendations for skin health.
- The Malnutrition Screening Tool (MUST) score^43^: a five-step assessment using body mass index (BMI), weight loss and nutritional intake, where a cumulative score $\ge$2 indicates high risk of malnutrition.

### Outcomes

The primary outcome was the total received in-hospital healthcare contacts, defined via documented nursing and rehabilitation inputs during hospital stay. Secondary endpoints were in-hospital death, extended hospital stay (defined as ≥14 days), non-home discharge, admission under older adult specialist care and requirement for rehabilitation (any rehabilitation-coded contact). All patient outcomes were evaluated up to hospital discharge. To assess change in performance and risk trajectory, we provide predictions of these outcomes using data available up to 5 distinct timepoints: ED arrival, at hospital admission, and 24, 48 and 72 h post-admission. Predictions at ED arrival were defined as received healthcare contacts within the index hospital stay.

### Data preprocessing for model development

**Fig. 4:**
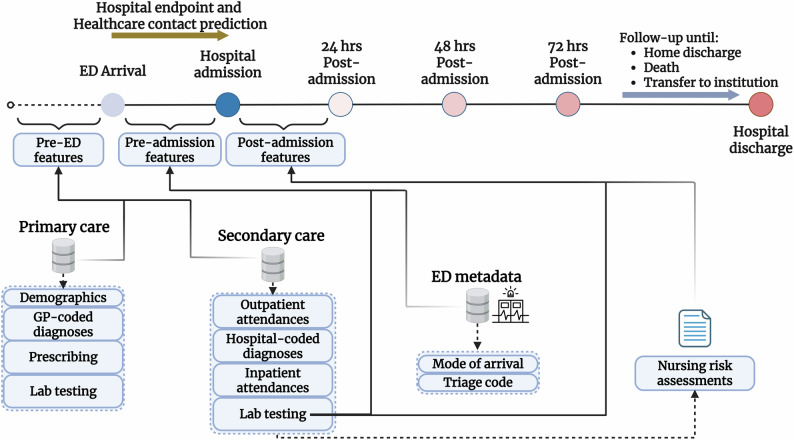
Flow diagram detailing the feature collection windows and individual prediction timepoints used to forecast healthcare contacts and related hospital outcomes. Predictions at the point of ED arrival are based on historical data from primary and secondary care. Predictions at the point of hospitalisation included available information collected during ED attendance. Predictions at 24 h post-admission and beyond further include routine nursing assessments and in-hospital lab testing within the target episode. Created in BioRender. Georgiev, K. (2025) https://BioRender.com/205crtq.

We adopted a data-driven approach to extracting and selecting the relevant features for prediction. Figure 4 details the clinical variables extracted to train a supervised ML model across each prediction time point. Only data available up to the point of prediction were included in each model. Apart from the test value, temporal variables describing the days since test collection, along with the 365-day average and standard deviations, were used as additional feature inputs. Morbidity features were generated based on the total number of previously diagnosed long-term conditions among the list of 25 phenotype groups. Additional binary variables categorised physical-mental multimorbidity (at least 1 physical and 1 mental condition), simple multimorbidity (2 or more conditions of any type) and high-count multimorbidity (4 or more conditions of any type). Outpatient visits were categorised by total number of attendances by specialty code, including feature variants for non-attendances. All categorical data were one-hot-encoded by adding ‘dummy’ binary variables for each possible option. Prescription features were counted by ATC group. As the healthcare contacts variable contained significant skewness, we used a bounded Box-Cox transformation at the point of prediction^44^, minimising the negative log-likelihood function of the contacts distribution to empirically identify the ideal scalar parameter $\lambda$. As the optimal $\lambda$ was estimated to be in the range (−0.3; 0.3), this was effectively equivalent to the natural logarithm of the contacts data. Further details on the classification of all extracted clinical variables, thresholds and temporal criteria can be found in Supplementary Table 1.

Data missingness was addressed by introducing a ‘meaningless’ value of ‘−1’, where appropriate for categorical and continuous data and ‘−9999’, where ‘−1’ was a possible valid measurement. This can effectively encourage non-linear models to learn how to ignore features with significant missingness^45^. Data cleaning procedures removed sparse features with <1% completeness within the collection window. We reported the relevant missingness across the top-ranked predictors for in-hospital healthcare contacts, grouped by population-specific hospital endpoints (admission to older adult specialist services and rehabilitation), shown in Supplementary Table 2.

### Feature selection procedures

Correlated features were removed using Pearson’s correlation coefficient with a threshold ≥0.9, determining significant linear relationships. We employed an additional cross-outcome feature selection procedure to filter meaningful predictor variables using the Boruta algorithm^46^. Boruta operates by generating random permutations called ‘shadow’ features, followed by training a Random Forest^47^ model to highlight the original features that scored higher than the most prominent ‘shadow’ features. Those features will be retained and marked as ‘important’. This process was repeated for 100 iterations, generating one importance set per outcome, where the union across all sets was used as the target feature set for training. Features marked as ‘tentative’ (the Boruta algorithm did not converge to a decision) were included in the importance set if the detected ranking score was ≤10 (among the top 10 tentative features for at least one outcome). The selected features used for model training are highlighted in Supplementary Table 1.

### Machine learning procedures

**Fig. 5:**
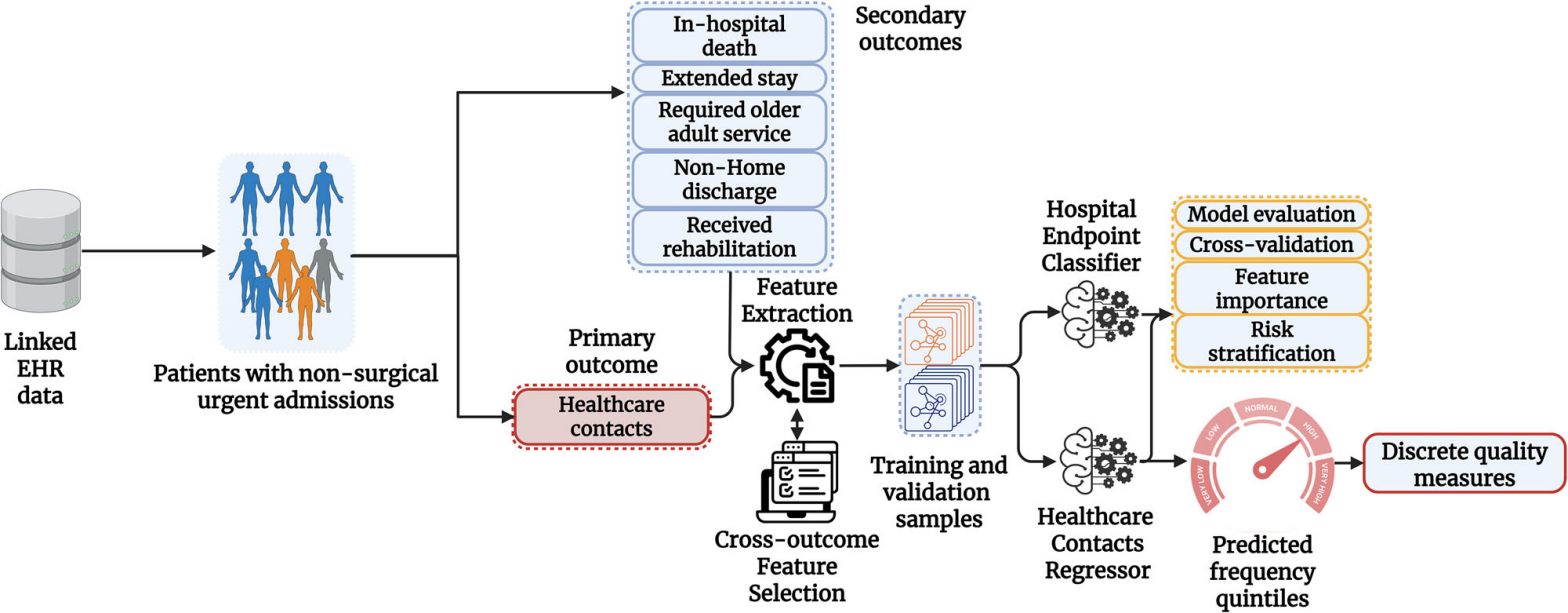
Overview of the data preprocessing, training and evaluation strategy. Created in BioRender. Georgiev, K. (2025) https://BioRender.com/b80mij6.

We used an ensemble model with gradient-boosted trees (XGBoost^48^) to develop a regression estimator for in-hospital healthcare contacts and a set of five classification estimators for each secondary outcome (data pipeline shown in Fig. 5). To train the models, we used a random stratified split (70% for training and 30% held-out for validation), balanced for age, sex, SIMD, total health contacts and each secondary endpoint, where applicable. In addition to a hold-out validation set, we adopted a leave-one-hospital-out validation strategy, evaluating model performance on independent data partitions across the three hospital sites. We further used a 10-fold stratified cross-validation during training to evaluate performance on random data partitions.

For the regression estimator, we reported performance using the MAE and cMAPE^49^, estimated after masking 0 values within the health contacts variable. MAPE is undefined (division by zero) if any actual predicted value is zero, and is heavily biased towards infinity for small actual values. Therefore, we computed a ‘conditional’ MAPE by excluding data points where the actual value is zero. We used absolute error measures as they are less sensitive to outliers compared to squared error estimates. Percentage errors like cMAPE additionally provide an interpretable and scale-independent measure suitable for forecasting tasks^49,50^.

To accurately measure model agreement, we equally discretised the prediction set and the original data within the validation set to define a five-level categorical variable describing the level of contact frequency: very low (VL), low (L), medium (M), high (H) and very high (VH) contacts. We then measured the Balanced Accuracy (BACC) Score across categories and the inter-rater reliability using the CKS. According to guidelines by Landis and Koch^51^, a CKS of 0 indicates agreement by random chance, with estimates closer to 1 indicating a more reliable systematic assessment. According to the guidelines, values up to 0.2 indicate almost no agreement, whereas values within [0.21–0.39], [0.40–0.59], [0.60–0.79], [0.80–0.90], [0.90–1] indicate minimal, weak, moderate, strong and ideal agreement, respectively. CKS was included as a chance-corrected agreement measure, which mitigates the inflation of agreement in imbalanced classes previously applied in clinical reliability studies^52^. The BACC is evaluated over five classes, and classification by random chance is set at 0.20, with estimates over this threshold indicating better than average sensitivity. We additionally used BACC, which explicitly accounts for unequal class sizes by averaging sensitivity and specificity, providing a fairer performance estimate in rarer contact groups^53^.

For the classification estimators, we reported the ROC-AUC and the PR-AUC, measuring overall discrimination ability and error rate for positive events, respectively. For PR-AUC, a higher score than the baseline outcome prevalence is treated as ‘better than random choice’. We additionally measured model sensitivity and specificity selected using the maximum F1-Score achieved for predicting an adverse event. We used decile-based discretisation on the probability scores to generate equally-sized risk groups, testing response rates within patients at the top 10% of predicted risk in the validation set. The DeLong method was used to generate 95% confidence intervals (95% CI) for all performance estimates, using a non-parametric algorithm to compute covariance between false positive rates, optimised for large sample sizes^54^. Details on the hyperparameter setup and training procedures are provided in the Supplementary Methods section. Hyperparameter tuning was performed using a grid search strategy focused on refining the maximum tree depth, learning rate and positive class weight of the XGBoost algorithms.

We reported the top 20 predictors and displayed their feature importances for estimating healthcare contacts using the TreeSHAP framework (Shapley Additive eXplanations)^55^. The aggregated Shapley values were estimated using the internal validation set of each prediction time point, summarised in global-level density plots. These values provided risk contribution scores, indicating the level of impact on contacts prediction.

Models were developed using Python version 3.10.12, using the ‘xgboost’ package (version 2.0.3) for training, ‘scikit-learn’ (version 1.3.2) for validation procedures and ‘shap’ (version 0.46.0) for feature importance analysis.

### Modelling setup and training strategy

All XGBoost models were trained over a maximum of 20,000 rounds with up to 100 early stopping rounds (patience threshold for the target loss function indicating when the model should interrupt its training). Hyperparameter tuning was conducted using a grid search strategy over an exponentially spaced grid for the learning rate and a linear grid for the maximum tree depth and any additional regularisation parameters. All classification estimators were trained with an additional prevalence weighting parameter (scale_pos_weight) based on the absolute ratio of patients with the outcome. The objective for optimisation was minimising the logistic loss, due to its convex nature and subsequent ability to produce well-calibrated probabilities in the presence of imbalanced data. The regression estimator used an objective function for minimising the pseudo-huber error, which balanced the properties of MAE and RMSE. Compared to the standard Huber loss, this modified loss provides a smoother approximation in gradient-based functions, making it less sensitive to the outliers present in the health contacts variable^56^.

### Statistical and risk analysis

Baseline patient characteristics were reported by the contact frequency group. Statistical testing was performed using the Kruskal–Wallis *H* test in non-normally distributed data and the one-way analysis of variance (ANOVA) test in normally distributed continuous data. Statistical testing in categorical variables was reported using Pearson’s chi-squared test. When reporting the spread of healthcare contacts by each secondary outcome, we used a two-sided Mann–Whitney–Wilcoxon test with Bonferroni correction. Statistical significance was assumed at *p* < 0.001. We provided additional survival analysis using an Aalen-Johansen estimator examining competing risks between in-hospital death and non-home discharge stratified by contact frequency level^57^.

### Study approval and consent

All data from EHR and national registries were linked and de-identified by the DataLoch service (Edinburgh, United Kingdom) and analysed within a Secure Data Environment (reference: DL_2022_001). DataLoch enables access to de-identified extracts of healthcare data from the South-East Scotland region to approved applicants: https://dataloch.org/. The study was reviewed and received approval under delegated authority from a regional National Health Service Research Ethics Committee (REC 22/NS/0093) and Caldicott Guardian. Individual patient consent was not required.

## Supplementary information

Supplementary Information

## Data Availability

The data that support the findings of this study are not openly available due to reasons of sensitivity, but summary data can be provided by the corresponding author upon reasonable request. Specific elements of the analysis may be made available upon reasonable request to the corresponding author, subject to standard application and approval processes.

## Code availability

The code used to conduct the exploratory data analysis, model development and validation is publicly available on GitHub, accessible via: https://github.com/kgeorgiev42/RCHD-Geriatric-Risk-Analytics/tree/main/notebooks/geriatric_ml.
