# Supplementary material for: Machine learning-based predictions of healthcare contacts following emergency hospitalisation using electronic health records
Source: NPJ Digit Med. 2025 Dec 17;8:764. doi: 10.1038/s41746-025-02138-4 (PMC12711894; doi:10.1038/s41746-025-02138-4)
Supplement: Supplementary file 1 — Supplementary Information [file 41746_2025_2138_MOESM1_ESM.pdf]

SUPPLEMENT

Machine Learning-based Predictions of Healthcare Contacts Following Emergency Hospitalisation Using Electronic Health Records

Konstantin Georgiev[1], Dimitrios Doudesis[1], Joanne McPeake[3], Nicholas L Mills[1], Susan D Shenkin[2], Jacques D Fleuriot[4], Atul Anand[1]

[1] Institute of Neuroscience and Cardiovascular Research, Queen's Medical Research Institute, University of Edinburgh, EH16 4TJ, UK
[2] Ageing and Health Research Group and Advanced Care Research Centre, Usher Institute, Edinburgh BioQuarter, University of Edinburgh, EH16 4UX, UK
[3] The Healthcare Improvement Studies Institute, Department of Public Health and Primary Care, University of Cambridge, CB1 8RN, UK
[4] Artificial Intelligence and its Applications Institute, School of Informatics, University of Edinburgh, EH8 9AB, UK

Correspondence to: Konstantin Georgiev**,** University of Edinburgh, Institute of Neuroscience and Cardiovascular Research**,** Chancellor's Building**,** 49 Little France Crescent**,** Edinburgh, EH16 4SA**,** United Kingdom, Email: K.S.Georgiev@sms.ed.ac.uk

Table and Figure legends:

Supplementary Table 1. Feature definitions for prediction of hospital outcomes and healthcare contacts.
Supplementary Table 2. Summary of missingness across specialist outcomes within the top-ranking predictors of healthcare contacts.
Supplementary Table 3. Patient characteristics at baseline grouped by in-hospital death. **Supplementary**
Supplementary Table 4. Patient characteristics at baseline grouped by extended stay.
Supplementary Table 5. Patient characteristics at baseline grouped by home discharge. **Supplementary**
Supplementary Table 6. Patient characteristics at baseline grouped by admission to geriatric medicine services.
Supplementary Table 7. Summary of healthcare contacts distribution by each secondary outcome.
Supplementary Table 8. Performance comparison across different linear and non-linear regression estimators for predictions of healthcare contacts at point of ED attendance.
Supplementary Table 9. Summary of stratified training and validation set characteristics for healthcare contacts prediction at point of ED attendance.
Supplementary Table 10. Summary of fine-tuned model hyperparameters across all outcomes for prediction models at point of ED attendance.
Supplementary Table 11. Performance comparison using stratified 10-fold validation for the healthcare contacts prediction model at point of ED attendance.

Supplementary Fig. 1. The annual distribution of secondary outcomes over the full data collection window.
Supplementary Fig. 2. Socio-demographic characteristics categorised by age and deprivation in patients with each secondary hospital outcome.
Supplementary Fig. 3. Box-plot showing the spread of log-transformed healthcare contacts across patients with and without each secondary outcome.
Supplementary Fig. 4. Violin plot showing the spread of log-transformed contacts and length of stay per individual across each hospital site.
Supplementary Fig. 5. Violin plot showing the spread of log-transformed contacts and length of stay per individual grouped by season of ED attendance.
Supplementary Fig. 6. Confusion Matrix summary showing the percentage of correctly captured and misclassified examples after quintile-based discretisation of the predicted contacts.
Supplementary Fig. 7. Performance trajectory curves for healthcare contact prediction, stratified by age group.
Supplementary Fig. 8. Performance trajectory curves for healthcare contact prediction, stratified by deprivation level.
Supplementary Fig. 9. Aalen-Johansen cumulative incidence function of in-hospital death stratified by healthcare contact level, adjusted for non-home discharge outcomes.
Supplementary Fig. 10. Performance trajectory curves for healthcare contact prediction measured in survivors to discharge.
Supplementary Fig. 11. Performance trajectory curves for healthcare contact prediction validated in patients admitted during the COVID-19 lockdown period within the UK (26th Mar 2020 – 19th Jul 2021), using training data on patients admitted before this period.
Supplementary Fig. 12. Violin plot showing the distribution of log-transformed nursing and rehabilitation contacts per individual in the validation set, grouped by surviving discharge across the three main predictors within the nursing risk assessments: Waterlow score, 4AT Score and mobility (bathing dependence).
Supplementary Fig. 13. Violin plot showing the distribution of log-transformed nursing and rehabilitation contacts per individual in the validation set, grouped by the documentation of a nursing risk assessment.

Supplementary Tables and Figures

Supplementary Table 1. Feature definitions for prediction of hospital outcomes and healthcare contacts. Features were excluded from training after correlation either due to having strong linear correlation with another variable (PCC>0.9) or during cross-outcome feature selection using the Boruta algorithm, after being rejected as important across all outcomes. SIMD – Scottish Index for Multiple Deprivation, 4AT – 4 A's Test for delirium screening, MUST – Malnutrition Universal Screening Tool for identifying adults at risk of undernutrition, COPD – Chronic Obstructive Pulmonary Disease. PCC – Pearson's corrleation coefficient. HA – point of hospital admission; 24h PA, 48h PA, 72h PA – 24, 48 and 72 hours post-admission respectively.

Group	Data type	Temporal sampling criteria	Variable thresholds	Selected for trainng (Y/N)	Prediction timepoint
**Demographics**					
**Age**	Continuous	At start of ED episode	/	Y	All models
**Sex**	Categorical	At start of ED episode	1 [Female], 0 [Male]	Y	All models
**SIMD (Quntiles)**	Ordinal	At start of ED episode	/	Y	All models
**ED information**					
**Mode of arrival:**<br>NHS Lothian Bus<br>Emergency Ambulance<br>Public Transport<br>Private Transport<br>GP Ambulance<br>Walked<br>Unknown<br>Other	Categorical	At start of ED episode	/	Y	All models
**Season of admission**	Categorical	At start of ED episode	/	Y	All models
**Attending hospital**<br>RIE<br>WGH<br>SJH	Categorical	At start of ED episode	/	Y	All models
**Triage code**	Ordinal	At start of admission	/	Y	HA,<br>24h PA,<br>48h PA,<br>72h PA
**Health questionnaires**					
**4AT Score**	Continuous	Within 24hrs of admission	/	Y	24h PA,<br>48h PA,<br>72h PA
**MUST Score**	Continuous	Within 24hrs of admission	/	Y	24h PA,<br>48h PA,<br>72h PA
**Waterlow Score**	Continuous	Within 24hrs of admission	/	Y	24h PA,<br>48h PA,<br>72h PA

**Bowel movement**<br>Urinary catheterisation<br>Urinary incontinence<br>Dysuria<br>Movement >6 times per day<br>Nocturia >2 times per night<br>Faeces incontinence<br>Constipation<br>Diarrhoea<br>Blood in stool<br>Bowel movement medication	Categorical	Within 24hrs of admission	/	Y	24h PA, 48h PA, 72h PA
**Falls assessment**<br>Clinical risk of falls<br>Fall within 6 months of admission	Categorical	Within 24hrs of admission	/	Y	24h PA, 48h PA, 72h PA
**Nutritional assessment**<br>Swallowing difficulties<br>Food allergies	Categorical	Within 24hrs of admission	/	Y	24h PA, 48h PA, 72h PA
**MRSA-related**<br>Infection prevention measures<br>Transfer with norovirus<br>Respiratory issues<br>Rash, fever or flu<br>Contact with infectious disease	Categorical	Within 24hrs of admission	/	Y	24h PA, 48h PA, 72h PA
**Rationale for use of bedrails**<br>Nursing fall risk assessment<br>At risk of bed-related fall	Categorical	Within 24hrs of admission	/	Y	24h PA, 48h PA, 72h PA
**Mobility**<br>Walking assistance<br>Bathing assistance<br>Toileting assistance<br>Bed rolling assistance<br>Moving up bed assistance<br>Out-of-bed assistance<br>Getting in bed assistance<br>Sit-stand-sit assistance<br>Lateral movement assistance<br>Floor-up assistance	Categorical	Within 24hrs of admission	Classified as independent, requiring assistance or not applicable due to bed rest	Y	24h PA, 48h PA, 72h PA
**Lab tests**					
eGFR (ml/min/1.73m^2),<br>Potassium (mEq/L),<br>Creatinine (mg/dL),<br>Urea (mg/dL),<br>Sodium (mEq/L),<br>Calcium (mmol/L),<br>TCO2 (pH),<br>Chloride (mmol/L),<br>Lymphocyte count (x10^9/L),<br>MCH (pg/cell),<br>Basophil count (x10^9/L),<br>Haematocrit (L/L),<br>Monocyte count (x10^9/L),<br>Eosinophil count (x10^9/L),<br>Neutrophil count (x10^9/L),<br>Red cell count (x10^9/L),<br>MCV (μm3),<br>Platelet count (x10^9/L),<br>White cell count (x10^9/L),	Continuous (test value), Categorical (abnormal-low, abnormal-high), Temporal (mean and std within previous 365 days)	Any point prior to prediction stage	0 [normal], 1 [above or below reference range]	Y<br>N<br>Y<br>Y<br>Y<br>Y<br>Y<br>N<br>Y<br>N<br>Y<br>Y<br>Y<br>Y<br>Y<br>Y<br>Y<br>Y<br>Y	All models

Haemoglobin (g/dl),<br>MCHC (g/dl),<br>C-reactive protein (mg/L),<br>Lactate (mmol/L),<br>ESR (mm/hr),<br>Ferritin (µg/L),<br>Bilirubin (µmol/L),<br>Albumin (g/dL),<br>Alkaline Phoshpatase (U/L),<br>ALT (IU/L),<br>GGT (IU/L),<br>AST (IU/L),<br>Glucose (mmol/L),<br>HbA1c (IFCC, mmol/mol),<br>Creatine Kinase (IU/L),<br>NT-proBNP (pg/mL),<br>Procalcitonin (ng/mL),<br>HS troponin I (ng/L),<br>HS troponin T (ng/L)				Y<br>N<br>Y<br>Y<br>Y<br>Y<br>Y<br>Y<br>Y<br>Y<br>Y<br>Y<br>N<br>Y<br>Y<br>N<br>N<br>Y<br>Y	
**Prescriptions**					
Bone metabolism related,<br>Antiplatelet drugs,<br>Diuretics,<br>Anti-hypertension,<br>Nitrates and CCBs,<br>Nausea and vertigo drugs,<br>Beta blockers,<br>Genito-urinary drugs,<br>Anticoagulants and protamine,<br>Parkinsonism-related,<br>Lipid regulators,<br>Anti-psychotics,<br>Anti-dementia drugs,<br>Antidepressants,<br>Total unique drug categories,	Continuous (# unique categories), Continuous (# prescribed by drug group)	Any point prior to prediction stage	/	Y,<br>Y,<br>Y,<br>Y,<br>Y,<br>Y,<br>Y,<br>N,<br>Y,<br>Y,<br>Y,<br>Y,<br>Y,<br>Y,<br>Y	All models
**Outpatient/inpatient attendances (by specialty)**					
General Medicine,<br>Cardiology,<br>Endocrinology,<br>Diabetes,<br>Gastroenterology,<br>Geriatric Medicine,<br>Medical Oncology,<br>Renal Medicine,<br>Neurology,<br>Palliative Medicine,<br>Rehabilitation Medicine,<br>Respiratory Medicine,<br>Rheumatology,<br>General Surgery,<br>Non-vascular Surgery,<br>Vascular Surgery,<br>Anaesthetics,<br>Cardiac Surgery,<br>Thoracic Surgery,<br>Ear/nose/throat,<br>Neurosurgery,<br>Opthalmology,	Continuous (# total attendances), Continuous (# attendances by specialty), Continuous (# failed attendances by specialty)	Any point prior to ED episode	/	Y<br>Y<br>Y<br>Y<br>Y<br>Y<br>Y<br>Y<br>Y<br>N<br>Y<br>Y<br>Y<br>Y<br>Y<br>N<br>Y<br>Y<br>N<br>Y<br>N	All models

Trauma/Orthopaedic Surgery,<br>Urology,<br>Oral Surgery,<br>Oral Medicine,<br>Clinical Oncology,<br>Haematology,<br>Infectious Diseases,<br>Dermatology,<br>Maxifollical Surgery,<br>Plastic Surgery,<br>Gynaecology,<br>General Psychiatry,<br>Psychiatry of Old Age,<br>Learning Disability,<br>Physiotherapy<br>Podiatry,<br>Dietetics,<br>Orthotics,<br># attendances in last 365 days,<br>Days since last attendance				Y<br>Y<br>Y<br>N<br>Y<br>Y<br>N<br>N<br>N<br>N<br>Y<br>Y<br>Y<br>Y<br>N<br>Y<br>N<br>N<br>N<br>Y<br>Y	
**Comorbidity history (SMR/GP READ coded)**					
Historical or Active cancer,<br>Liver disease,<br>Atrial fibrillation,<br>Chronic renal disease,<br>Obesity,<br>Hip fracture,<br>Bronchiectasis,<br>Diabetes,<br>COPD,<br>Chronic psychiatric disorder**,**<br>Progressive neurological disease†,<br>Inflammatory bowel disease,<br>Osteoporosis,<br>Aortic aneurysm,<br>Stroke,<br>Ischaemic heart disease,<br>Epilepsy,<br>Hypertension,<br>Spinal stenosis,<br>Heart failure,<br>Alcohol/substance misuse,<br>Arthritis/other arthropathy,<br>Asthma,<br>Depression,<br>Peripheral vascular disease,<br>Pulmonary fibrosis,<br># long-term conditions,<br># physical long-term conditions,<br># mental long-term conditions<br>Physical-mental multimorbidity	Categorical (Presence/absence of previous diagnosis), Temporal (days since last diagnosis), Continuous (unique physical/mental conditions)	Any point prior to ED episode	/	Y<br>Y<br>Y<br>Y<br>Y<br>Y<br>N<br>Y<br>Y<br>Y<br>Y<br>Y<br>Y<br>N<br>Y<br>Y<br>Y<br>Y<br>N<br>Y<br>Y<br>Y<br>Y<br>Y<br>Y<br>Y<br>Y<br>Y<br>Y<br>Y	All models

†Condition group includes diagnoses of dementia.

Supplementary Table 2. Summary of missingness across specialist outcomes within the top-ranking predictors of healthcare contacts. Values are displayed total and % missing measurements per patient across the study population.

	All (n=98242)	Outcome	
		Geriatric Medicine services (n=13301)	Received rehabilitation* (n=40946)
**SIMD**	970 (1%)	61 (1%)	294 (1%)
**ED variables**			
**Mode of arrival**	6469 (7%)	295 (2%)	1551 (4%)
**Triage code**	5242 (5%)	216 (2%)	1208 (3%)
**Nursing assessments**			
**4AT Score**	43633 (44%)	5160 (39%)	17098 (42%)
**Waterlow Score**	20314 (21%)	901 (7%)	5086 (12%)
**Bathing dependence**	54885 (56%)	5705 (43%)	19736 (48%)
**Floor-up moving assistance**	54436 (55%)	5509 (41%)	19282 (47%)
**Lab test values**			
**Albumin (g/L)**	26706 (27%)	3124 (24%)	11041 (27%)
**Bilirubin (umol/L)**	7821 (8%)	1389 (10%)	4276 (10%)
**Urea (mmol/L)**	9169 (9%)	1534 (12%)	4779 (12%)
**Lymphocyte count**	7430 (7%)	1363 (10%)	4185 (10%)
**Basophil count**	7632 (8%)	1416 (11%)	4305 (11%)
**Eosinophil count**	8628 (9%)	1648 (12%)	4833 (12%)
**Neutrophil count**	7429 (7%)	1362 (10%)	4184 (10%)
**Erythrocyte Sedimentation Rate (mm/hr)**	59455 (61%)	7816 (59%)	23695 (58%)
**Alkaline Phosphatase (U/L)**	7853 (8%)	1390 (11%)	4288 (11%)
**Alanine Transaminase (U/L)**	7853 (8%)	1394 (11%)	4290 (11%)
**HS Troponin T (ng/L)**	92508 (94%)	13007 (98%)	39569 (97%)
**HS Troponin I (ng/L)**	76455 (78%)	11723 (88%)	34986 (85%)
**Creatine Kinase (U/L)**	84524 (86%)	10006 (75%)	33392 (82%)
**estimated Glomerular Filtration Rate (ml/min/1.73m²)**	7197 (7%)	4088 (10%)	1337 (10%)
**C-reactive Protein (mg/L)**	18697 (19%)	2410 (18%)	8429 (21%)
**Gamma-Glutamyl Transferase (U/L)**	26532 (27%)	3738 (28%)	11773 (29%)
**Sodium (mmol/L)**	7222 (7%)	1330 (10%)	4093 (10%)

*Defined as physiotherapy, occupational therapy or speech and language therapy.
SIMD – Scottish Index for Multiple Deprivation.

Supplementary Table 3. Patient characteristics at baseline grouped by in-hospital death. Values are displayed in patient counts (%) unless stated otherwise. Statistical testing: One-way analysis of variance (ANOVA) test in numerical data, Chi-squared test in categorical data. SIMD – Scottish Index for Multiple Deprivation, 4AT – 4 A's Test for delirium screening, MUST – Malnutrition Universal Screening Tool for identifying adults at risk of undernutrition.

	All (n=98242)	In-hospital death		p
		Y (n=6093)	N (n=92149)	
**Age (mean, SD)**	72 (12)	78 (11)	71 (12)	<0.001
**Women**	50214 (51%)	3017 (49%)	47197 (51%)	0.01
**SIMD in quintiles**				0.16
**1 (most deprived)**	15735 (16%)	924 (15%)	14811 (16%)	
**2-4**	57608 (59%)	3597 (59%)	54011 (59%)	
**5 (least deprived)**	24899 (25%)	1572 (26%)	23327 (25%)	
**Attending hospital**				<0.001
**RIE**	49891 (50%)	3233 (53%)	46658 (51%)	
**WGH**	29940 (31%)	1044 (17%)	17367 (19%)	
**SJH**	18411 (19%)	1816 (30%)	28124 (31%)	
**Medical condition history**				
**# Long-term conditions (median, IQR)**	3 [2, 5]	4 [3, 6]	3 [2, 5]	<0.001
**Simple Multimorbidity (≥2 conditions)**	33534 (34%)	1830 (30%)	31704 (34%)	<0.001
**High-count Multimorbidity (≥4 conditions)**	48655 (50%)	3707 (61%)	44948 (49%)	<0.001
**Physical-mental Multimorbidity (≥1 physical and ≥1 mental condition)**	36096 (37%)	2016 (33%)	34080 (37%)	<0.001
**Health questionnaire results**				
**4AT Score (≥4, at risk)**	6540 (7%)	1519 (25%)	5021 (5%)	<0.001
**MUST Score (≥2, at high risk)**	5911 (6%)	1150 (19%)	4761 (5%)	<0.001
**Waterlow score (≥10, at risk)**	17023 (17%)	1477 (24%)	15546 (17%)	<0.001
**Fall event within 6 months of admission**	16043 (16%)	1481 (24%)	14562 (16%)	<0.001
**Walking dependence**	17074 (21%)	1121 (18%)	15953 (17%)	<0.001
**Bathing dependence**	20160 (21%)	2086 (34%)	18074 (20%)	<0.001
**Swallowing difficulties**	1719 (2%)	418 (7%)	1301 (1%)	<0.001

Supplementary Table 4. Patient characteristics at baseline grouped by extended stay. Values are displayed in patient counts (%) unless stated otherwise. Statistical testing: One-way analysis of variance (ANOVA) test in numerical data, Chi-squared test in categorical data. SIMD – Scottish Index for Multiple Deprivation, 4AT – 4 A's Test for delirium screening, MUST – Malnutrition Universal Screening Tool for identifying adults at risk of undernutrition.

	All (n=98242)	Extended stay (≥14 days)		p
		Y (n=19040)	N (n=79202)	
**Age (mean, SD)**	72 (12)	78 (11)	70 (12)	<0.001
**Women**	50214 (51%)	10,624 (56%)	39,590 (50%)	<0.001
**SIMD in quintiles**				<0.001
**1 (most deprived)**	15735 (16%)	2802 (15%)	12933 (16%)	
**2-4**	57608 (59%)	10931 (57%)	46677 (59%)	
**5 (least deprived)**	24899 (25%)	5307 (28%)	19592 (25%)	
**Attending hospital**				<0.001
**RIE**	49891 (50%)	10025 (53%)	39866 (50%)	
**WGH**	29940 (31%)	2885 (15%)	15526 (20%)	
**SJH**	18411 (19%)	6130 (32%)	23810 (30%)	
**Medical condition history**				
**# Long-term conditions (median, IQR)**	3 [2, 5]	4 [3, 6]	3 [2, 5]	<0.001
**Simple Multimorbidity (≥2 conditions)**	33534 (34%)	5997 (32%)	27537 (35%)	<0.001
**High-count Multimorbidity (≥4 conditions)**	48655 (50%)	11056 (58%)	37599 (48%)	<0.001
**Physical-mental Multimorbidity (≥1 physical and ≥1 mental condition)**	36096 (37%)	6709 (35%)	29387 (37%)	<0.001
**Health questionnaire results**				
**4AT Score (≥4, at risk)**	6540 (7%)	2629 (14%)	3911 (5%)	<0.001
**MUST Score (≥2, at high risk)**	5911 (6%)	2635 (14%)	3276 (4%)	<0.001
**Waterlow score (≥10, at risk)**	17023 (17%)	5961 (31%)	11062 (14%)	<0.001
**Fall event within 6 months of admission**	16043 (16%)	5990 (31%)	10053 (13%)	<0.001
**Walking dependence**	17074 (21%)	5327 (28%)	11747 (15%)	<0.001
**Bathing dependence**	20160 (21%)	7867 (41%)	12293 (16%)	<0.001
**Swallowing difficulties**	1719 (2%)	957 (5%)	762 (1%)	<0.001

Supplementary Table 5. Patient characteristics at baseline grouped by home discharge. Values are displayed in patient counts (%) unless stated otherwise. Statistical testing: One-way analysis of variance (ANOVA) test in numerical data, Chi-squared test in categorical data. SIMD – Scottish Index for Multiple Deprivation, 4AT – 4 A's Test for delirium screening, MUST – Malnutrition Universal Screening Tool for identifying adults at risk of undernutrition.

	**All (n=98242)**	**Home discharge**		**p**
		**Y (n=85904)**	**N (n=12338)**	
**Age (mean, SD)**	72 (12)	71 (12)	78 (11)	<0.001
**Women**	50214 (51%)	43752 (51%)	6462 (52%)	0.03
**SIMD in quintiles**				<0.001
**1 (most deprived)**	15735 (16%)	13910 (16%)	1825 (15%)	
**2-4**	57608 (59%)	50446 (59%)	7162 (58%)	
**5 (least deprived)**	24899 (25%)	21548 (25%)	3351 (27%)	
**Attending hospital**				<0.001
**RIE**	49891 (50%)	43122 (50%)	6769 (55%)	
**WGH**	29940 (31%)	16397 (19%)	2014 (16%)	
**SJH**	18411 (19%)	26385 (31%)	3555 (29%)	
**Medical condition history**				
**# Long-term conditions (median, IQR)**	3 [2, 5]	4 [3, 6]	3 [2, 5]	<0.001
**Simple Multimorbidity (≥2 conditions)**	33534 (34%)	3798 (31%)	29736 (35%)	<0.001
**High-count Multimorbidity (≥4 conditions)**	48655 (50%)	7275 (59%)	41380 (48%)	<0.001
**Physical-mental Multimorbidity (≥1 physical and ≥1 mental condition)**	36096 (37%)	4306 (35%)	31790 (37%)	<0.001
**Health questionnaire results**				
**4AT Score (≥4, at risk)**	6540 (7%)	3563 (4%)	2977 (24%)	<0.001
**MUST Score (≥2, at high risk)**	5911 (6%)	3953 (5%)	1958 (16%)	<0.001
**Waterlow score (≥10, at risk)**	17023 (17%)	13873 (16%)	3150 (26%)	<0.001
**Fall event within 6 months of admission**	16043 (16%)	12461 (15%)	3582 (29%)	<0.001
**Walking dependence**	17074 (21%)	14375 (17%)	2699 (22%)	<0.001
**Bathing dependence**	20160 (21%)	15421 (18%)	4739 (38%)	<0.001
**Swallowing difficulties**	1719 (2%)	943 (1%)	776 (6%)	<0.001

Supplementary Table 6. Patient characteristics at baseline grouped by admission to geriatric medicine services. Values are displayed in patient counts (%) unless stated otherwise. Statistical testing: One-way analysis of variance (ANOVA) test in numerical data, Chi-squared test in categorical data. SIMD – Scottish Index for Multiple Deprivation, 4AT – 4 A's Test for delirium screening, MUST – Malnutrition Universal Screening Tool for identifying adults at risk of undernutrition.

	**All (n=98242)**	**Admitted to Geriatric Medicine Services**		**p**
		**Y (n=13301)**	**N (n=84941)**	
**Age (mean, SD)**	72 (12)	83 (8)	70 (12)	<0.001
**Women**	50214 (51%)	8167 (61%)	42047 (50%)	<0.001
**SIMD in quintiles**				<0.001
**1 (most deprived)**	15735 (16%)	1657 (13%)	14078 (17%)	
**2-4**	57608 (59%)	7409 (56%)	50,99 (59%)	
**5 (least deprived)**	24899 (25%)	4235 (32%)	20664 (24%)	
**Attending hospital**				<0.001
**RIE**	49891 (50%)	8176 (62%)	41715 (49%)	
**WGH**	29940 (31%)	1357 (10%)	17054 (20%)	
**SJH**	18411 (19%)	3768 (28%)	26172 (31%)	
**Medical condition history^{+}**				
**# Long-term conditions (median, IQR)**	3 [2, 5]	4 [3, 6]	3 [2, 5]	<0.001
**Simple Multimorbidity (≥2 conditions)**	33534 (34%)	3934 (30%)	29600 (35%)	<0.001
**High-count Multimorbidity (≥4 conditions)**	48655 (50%)	8236 (62%)	40419 (48%)	<0.001
**Physical-mental Multimorbidity (≥1 physical and ≥1 mental condition)**	36096 (37%)	4130 (31%)	31966 (38%)	<0.001
**Health questionnaire results**				
**4AT Score (≥4, at risk)**	6540 (7%)	2523 (19%)	4017 (5%)	<0.001
**MUST Score (≥2, at high risk)**	5911 (6%)	1705 (13%)	4206 (5%)	<0.001
**Waterlow score (≥10, at risk)**	17023 (17%)	4433 (33%)	12590 (15%)	<0.001
**Fall event within 6 months of admission**	16043 (16%)	5375 (40%)	10668 (13%)	<0.001
**Walking dependence**	17074 (21%)	4485 (34%)	12589 (15%)	<0.001
**Bathing dependence**	20160 (21%)	6414 (48%)	13746 (16%)	<0.001
**Swallowing difficulties**	1719 (2%)	506 (4%)	1213 (1%)	<0.001

Supplementary Table 7. Summary of healthcare contacts distribution by each secondary outcome. Values are displayed as median [IQR] unless stated otherwise. Statistical testing: One-way analysis of variance (ANOVA) test in numerical data reported as mean (SD), Kruskal-Wallis H test in numerical data reported as median [IQR], Chi-squared test in categorical data. INH-D – In-hospital death, EXT-ST – Extended stay (≥14 days), NHM-D – Non-home discharge, ADM-GM – Admitted to geriatric medicine services.

	All (n=98242)	Secondary outcome (n, %)			
		INH-D (n=6093, 6%)	EXT-ST (n=19040, 19%)	NHM-D (n=12338, 13%)	ADM-GM (n=13301, 14%)
**Overall**					
**Health contacts per admission**+	7 [3, 19]	20 [8, 47]	51 [30, 90]	23 [8, 60]	31 [11, 70]
**Health contacts per admission day**+	2 [2, 4]	4 [2, 7]	7 [4, 12]	4 [2, 8]	5 [3, 9]
**Number of disciplines involved (mean, SD)**+	2 (1)	2 (1)	3 (1)	2 (1)	3 (1)
**Nursing**					
**Nursing contacts per admission**	5 [2, 12]	13 [6, 28]	28 [13, 51]	14 [5, 35]	14 [5, 36]
**Nursing contacts per admission day**	2 [1, 3]	3 [2, 4]	4 [2, 7]	3 [2, 5]	3 [2, 5]
**Rehabilitation**					
**Received any rehabilitation (n, %)**	40946 (42%)	3796 (62%)	17210 (90%)	8299 (67%)	11897 (89%)
**Rehabilitation contacts per admission***	6 [2, 17]	8 [3, 19]	19 [9, 36]	10 [4, 24]	14 [5, 30]
**Rehabilitation contacts per admission day***	2 [2, 4]	2 [2, 4]	4 [2, 6]	3 [2, 4]	3 [2, 5]
**Time to first rehabilitation contact (hours)***	42 [19, 90]	55 [22, 113]	61 [23, 131]	50 [22, 105]	42 [20, 85]

+Includes nursing and rehabilitation disciplines.
*Rehabilitation data were calculated only for those who received at least one rehabilitation contact (defined as physiotherapy, occupational therapy or speech and language therapy).

Supplementary Table 8. Performance comparison across different linear and non-linear regression estimators for predictions of healthcare contacts at point of ED attendance. The models were fine-tuned based on a grid search strategy using the listed hyperparameters. Solver – automatically selected using scikit-learn or based on Singular Value Decomposition. L1 ratio – mixing parameter for ElasticNet indicating the balance between L1 and L2 penalties for coefficients. Alpha – constant that multiplies penalty terms. Splitter – whether to include randomisation within tree splits. Max features – subsample based on sqrt or log2 of feature size. Criterion – MSE, MSE with Friedman's improvement score for potential splits or using reduction in the half mean Poisson deviance. RMSE – Root Mean Squared Error, MAE – Mean Absolute Error, cMAPE - conditional Mean Absolute Percentage Error with masked 0 values.

**Hyperparameters and performance summary**	**Model type**					
	**OLSR**	**Ridge**	**Decision Tree**	**ElasticNet**	**Random Forest**	**XGBoost**
**Parameter grid**	/	Fit intercept: {Y, N}; Solver: {'auto', 'svd'}	splitter: {'best', 'random'}; criterion: {'absolute error', 'Friedman MSE', 'Poisson'}; min samples per split: {2, 3, 5, 10}; max features: {sqrt, log2}	Fit intercept: {Y, N}; alpha: {0.001, 0.01, 0.1, 1}; L1 ratio: {0.001, 0.1, 0.5, 1}	# estimators: {5, 10, 25, 20}; criterion: {'absolute error', 'Friedman MSE', 'Poisson'}; min samples per split: {2, 3, 5, 10}; max features: {sqrt, log2}	Parameter setup from Supplementary Table 10
**Evaluation results**						
**RMSE**	1.21 [1.20, 1.22]	1.21 [1.20, 1.22]	1.50 [1.48, 1.51]	1.21 [1.20, 1.22]	1.19 [1.18, 1.20]	1.18 [1.17, 1.19]
**MAE**	0.96 [0.95, 0.97]	0.96 [0.95, 0.97]	1.18 [1.17, 1.19]	0.96 [0.95, 0.97]	0.95 [0.94, 0.96]	0.94 [0.93, 0.95]
**cMAPE (%)**	49.0% [48.4%, 49.7%]	49.0% [48.4%, 49.7%]	61.6% [60.8%, 62.5%]	49.0% [48.5%, 49.7%]	48.6% [48.1%, 49.3%]	46.9% [46.3%, 47.5%]

Supplementary Table 9. Summary of stratified training and validation set characteristics for healthcare contacts prediction at point of ED attendance. Values are in proportion of patients (%) unless stated otherwise. Statistical testing: Kruskal-Wallis H test in numerical data reported as median [IQR], Chi-squared test in categorical data. SIMD – Scottish Index for Multiple Deprivation.

**Characteristic**	**All (n=98,242)**	**Training (n=68,769)**	**Validation (n=29,743)**	**p**
**Age (median, IQR)**	73 (62, 81)	73 (62, 81)	73 (62, 81)	0.91
**Age group**				1.00
**50-59**	19508 (20%)	13655 (20%)	5853 (20%)	..
**60-69**	21154 (22%)	14807 (22%)	6347 (22%)	..
**70-79**	27637 (28%)	19347 (28%)	8290 (28%)	..
**80-89**	23981 (24%)	16787 (24%)	7194 (24%)	..
**90+**	5962 (6%)	4173 (6%)	1789 (6%)	..
**Women**	50214 (51%)	35151 (51%)	15063 (51%)	0.99
**SIMD in quintiles**				1.00
**1 (most deprived)**	15735 (16%)	11019 (16%)	4716 (16%)	..
**2-4**	57608 (59%)	40331 (59%)	17277 (59%)	..
**5 (least deprived)**	24899 (25%)	17419 (25%)	7480 (25%)	..
**Hospital outcomes**				
**In-hospital death**	6093 (6%)	4283 (6%)	1810 (6%)	0.62
**Extended stay (≥14 days)**	19040 (19%)	13344 (19%)	5696 (19%)	0.78
**Non-home discharge**	12338 (13%)	8622 (13%)	3716 (13%)	0.77
**Admission to Geriatric Medicine services**	13301 (14%)	9335 (14%)	3966 (13%)	0.56
**Received rehabilitation***	40946 (42%)	28663 (42%)	12283 (42%)	0.99
**Healthcare contacts**				
**Overall contacts per admission+**	4334 (3%)	3034 (3%)	1300 (3%)	0.91
**Rehabilitation contacts per admission*+**	8899 (6%)	6230 (6%)	2669 (6%)	0.49
**Number of disciplines involved+**				0.40
**1**	58063 (59%)	40564 (59%)	17499 (59%)	..
**2**	18135 (19%)	12673 (18%)	5462 (19%)	..
**3+**	22044 (23%)	15532 (23%)	6512 (22%)	..

+Includes nursing and rehabilitation disciplines.
*Rehabilitation data were calculated only for those who received at least one rehabilitation contact (defined as physiotherapy, occupational therapy or speech and language therapy).

Supplementary Table 10. Summary of fine-tuned model hyperparameters across all outcomes for prediction models at point of ED attendance. The huber loss combines the properties of the Mean Absolute Error (MAE) and Root Mean Squared Error (RMSE) during training. The pseudo-huber loss provides a smooth approximation to this error which is optimal for gradient-based learners, such as XGBoost. 'scale_pos_weight' is a constant scaling parameter applied during training to balance feature weights by outcome prevalence, estimated as the ratio: $\frac{\#\,participants\ without\ outcome}{\#\,participants\ with\ outcome}$. CINT – Care Intensity prediction model, INH-D – in-hospital death model, EXT-ST – extended hospital stay model, NHM-D – non-home discharge model, ADM-GM – admission to geriatric medicine services model, REHAB – rehabilitation classification model.

XGBoost model hyperparameter	Outcome					
	**CINT**	**INH-D**	**EXT-ST**	**NHM-D**	**ADM-GM**	**REHAB**
**Training rounds**	20,000	20,000	20,000	20,000	20,000	20,000
**Early stopping rounds**	50	100	100	100	50	100
**Objective function**	Pseudo-huber Error	Logistic Loss	Logistic Loss	Logistic Loss	Logistic Loss	Logistic Loss
**Max tree depth (max_depth)**	3	4	3	3	4	3
**Learning rate (eta)**	0.01	0.1	0.01	0.01	0.001	0.01
**Positive class weight (scale_pos_weight)**	/	10.0	4.2	6.9	6.4	1.4

Supplementary Table 11. Performance comparison using stratified 10-fold validation for the healthcare contacts prediction model at point of ED attendance. The model hyperparameter setup is provided in Supplementary Table 10. RMSE – Root Mean Squared Error, MAE – Mean Absolute Error, cMAPE - conditional Mean Absolute Percentage Error with masked 0 values.

Stratified K-fold evaluation	Fold #										
	1	2	3	4	5	6	7	8	9	10	Mean
RMSE	1.21 [1.20, 1.23]	1.23 [1.21, 1.25]	1.23 [1.21, 1.24]	1.23 [1.22, 1.25]	1.21 [1.20, 1.23]	1.22 [1.20, 1.24]	1.23 [1.21, 1.24]	1.23 [1.21, 1.25]	1.22 [1.20, 1.24]	1.22 [1.20, 1.23]	1.22±0.01
MAE	0.96 [0.95, 0.98]	0.97 [0.96, 0.99]	0.97 [0.95, 0.98]	0.98 [0.97, 0.99]	0.97 [0.95, 0.98]	0.97 [0.95, 0.98]	0.97 [0.96, 0.99]	0.98 [0.96, 0.99]	0.97 [0.96, 0.99]	0.97 [0.95, 0.98]	0.97±0.01
cMAPE (%)	48% [47-49%]	49% [48-50%]	49% [48-50%]	49% [48-50%]	49% [48-50%]	49% [48-50%]	48% [47-50%]	49% [48-50%]	49% [48-50%]	50% [49-51%]	49%±0.42

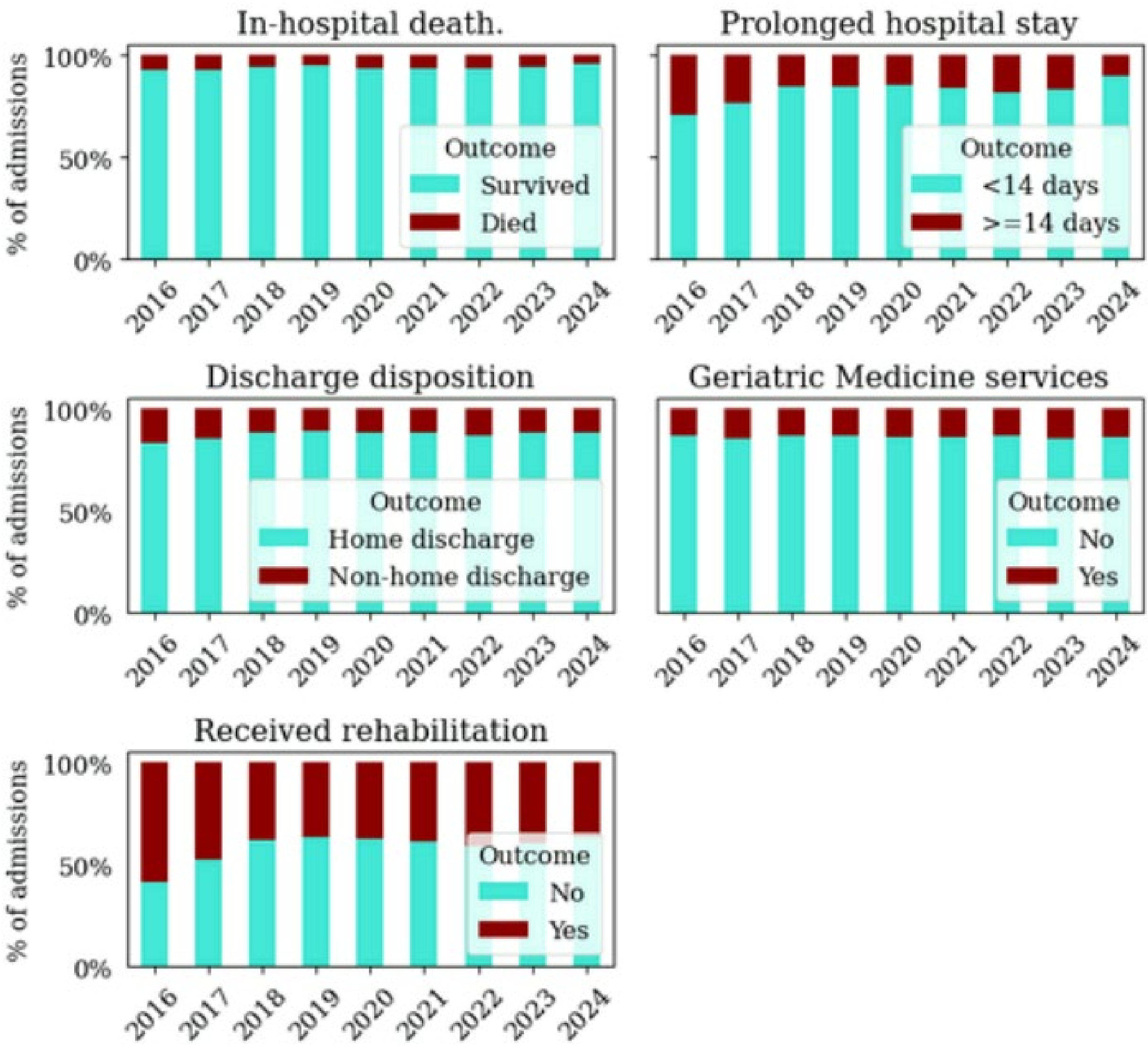

Supplementary Fig. 1. The annual distribution of secondary outcomes over the full data collection window.

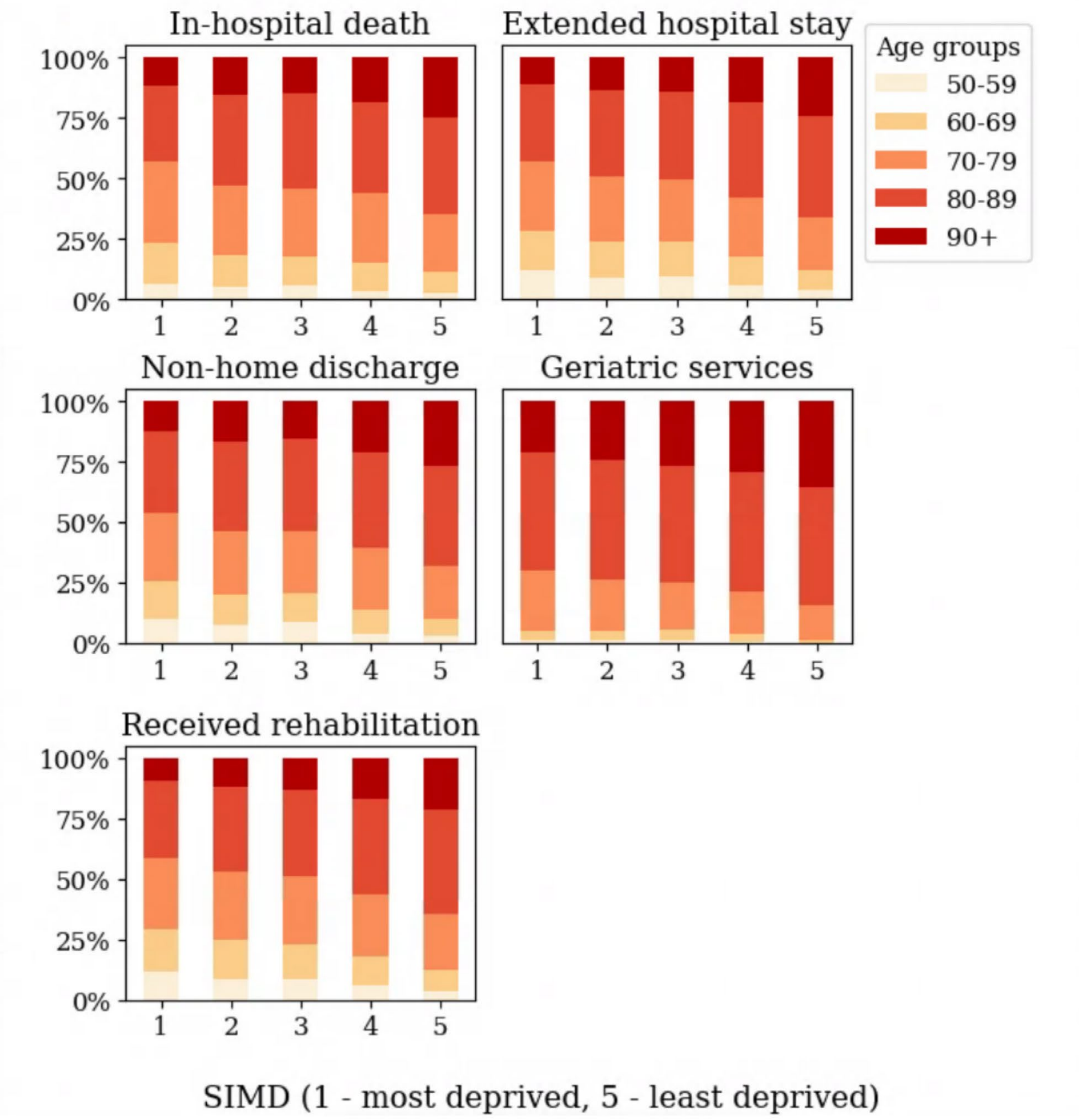

Supplementary Fig. 2. Socio-demographic characteristics categorised by age and deprivation in patients with each secondary hospital outcome. SIMD – Scottish Index for Multiple Deprivation (1 – most deprived, 5 – least deprived).

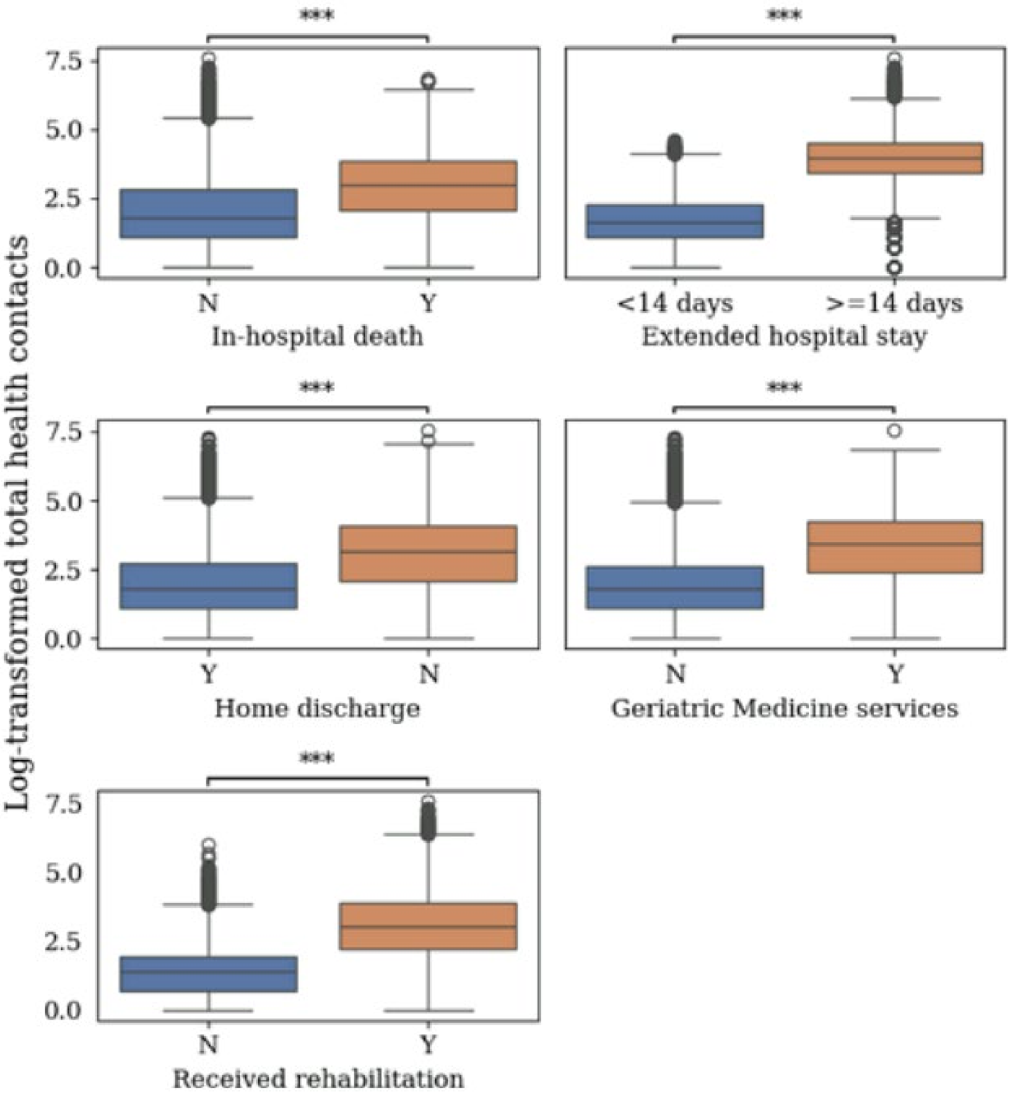

Supplementary Fig. 3. Box-plot showing the spread of log-transformed healthcare contacts across patients with and without each secondary outcome. Statistical testing: Two-sided Mann-Whitney-Wilcoxon test with Bonferroni correction with significance measured at $p<0.001$. U statistic values: In-hospital death (U=1.65e+08), Extended stay (U=1.09e+08), Home discharge (U=7.79e+08), Geriatric Medicine services (U=2.42e+08), Received rehabilitation (U=3.43e+08).

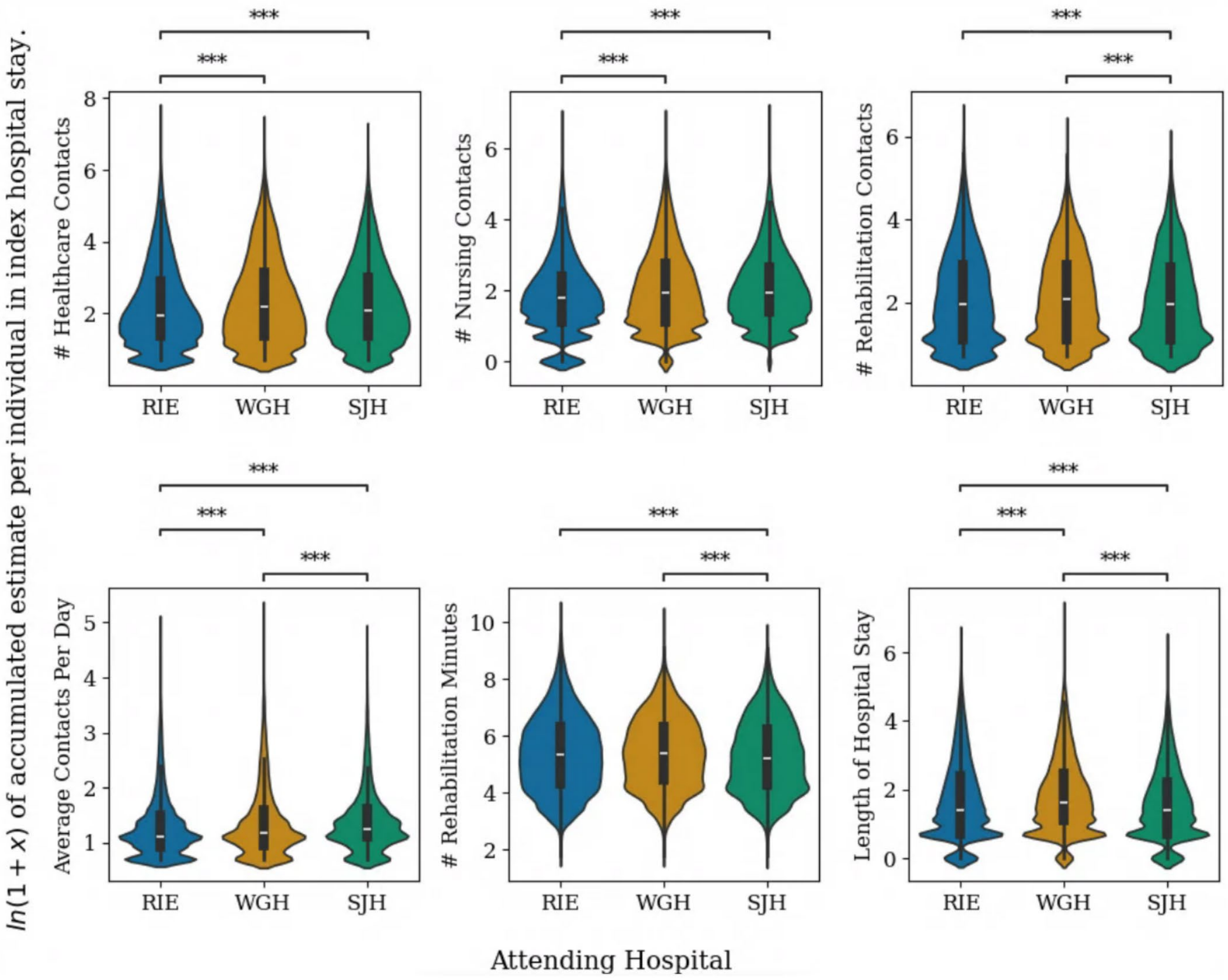

Supplementary Fig. 4. Violin plot showing the spread of log-transformed contacts and length of stay per individual across each hospital site. Statistical testing: Two-sided Mann-Whitney-Wilcoxon test with Bonferroni correction with significance presented at p<0.001. Hospital codes: RIE – Royal Infirmary of Edinburgh, WGH – Western General Hospital, SJH – Saint John's Hospital.

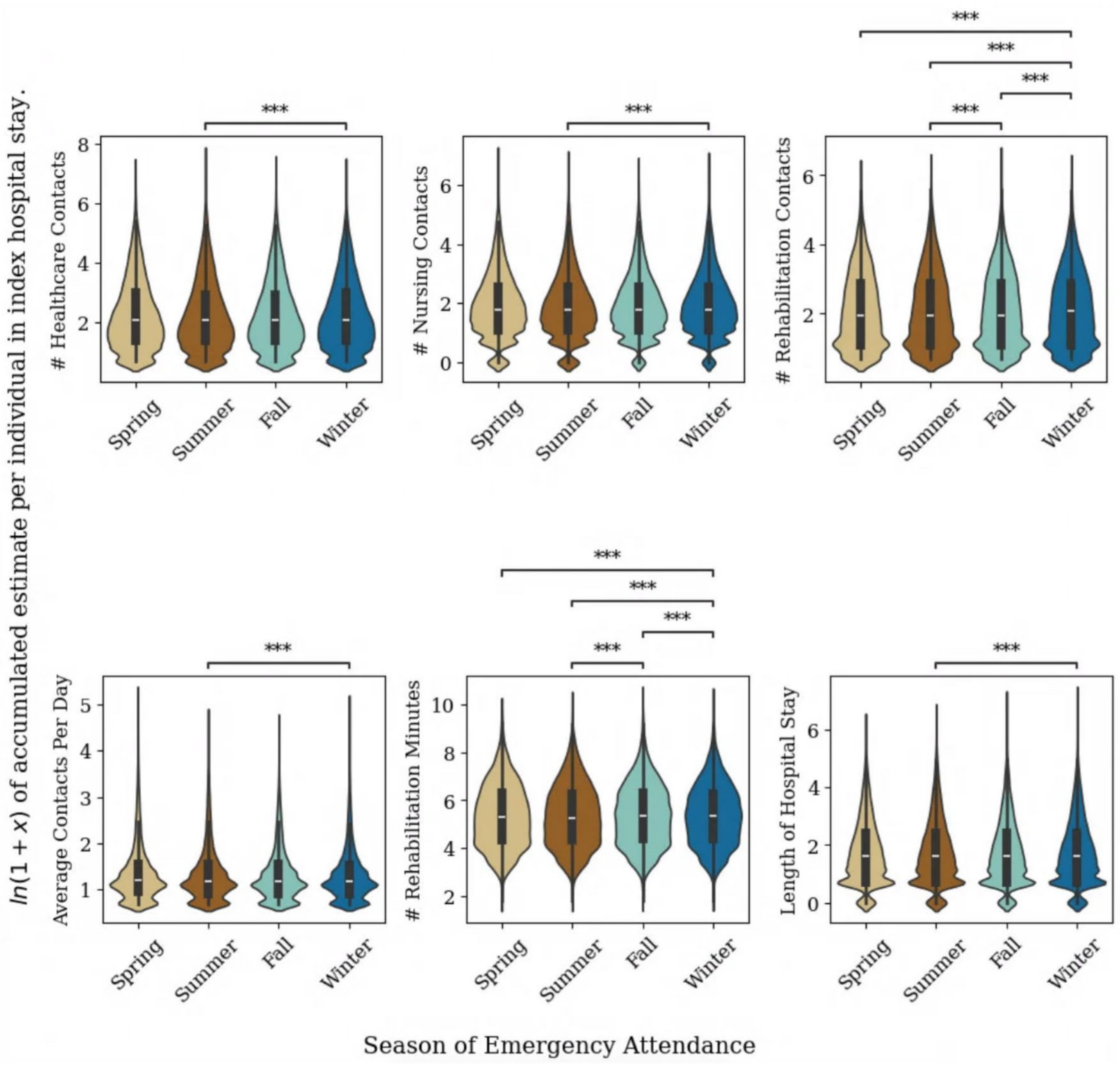

Supplementary Fig. 5. Violin plot showing the spread of log-transformed contacts and length of stay per individual grouped by season of ED attendance. Statistical testing: Two-sided Mann-Whitney-Wilcoxon test with Bonferroni correction with significance presented at $p<0.001$.

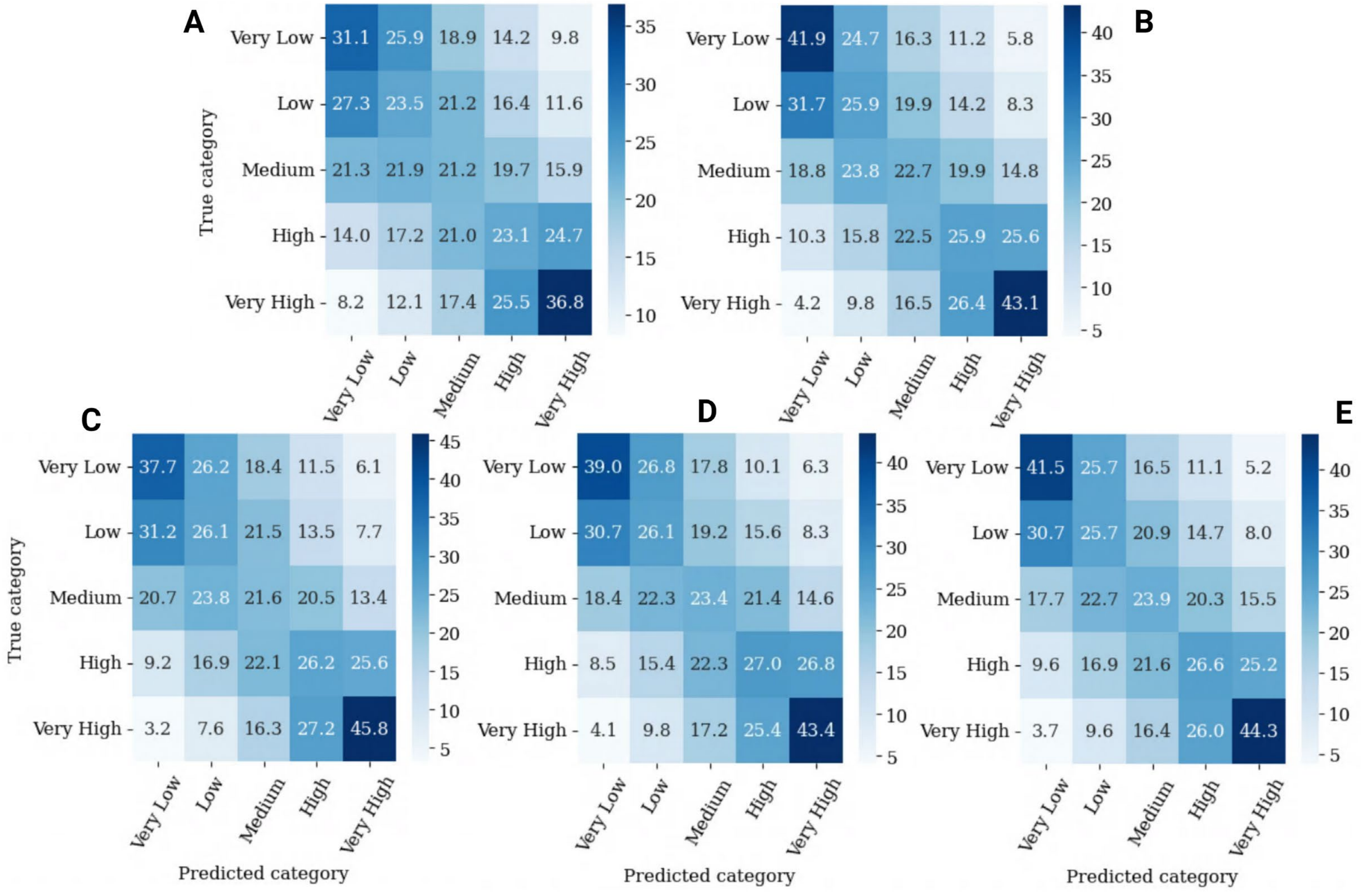

Supplementary Fig. 6. Confusion Matrix summary showing the percentage of correctly captured and misclassified examples after quintile-based discretisation of the predicted contacts. Categories shown on y-axis indicate true categories after binning the samples from the original data, while categories shown on x-axis indicate the binned samples of the predictions. (A)—Point of ED arrival, (B)—Point of hospital admission, (C)—24 hours post-admission, (D)—48 hours post-admission, (E)—72 hours post-admission.

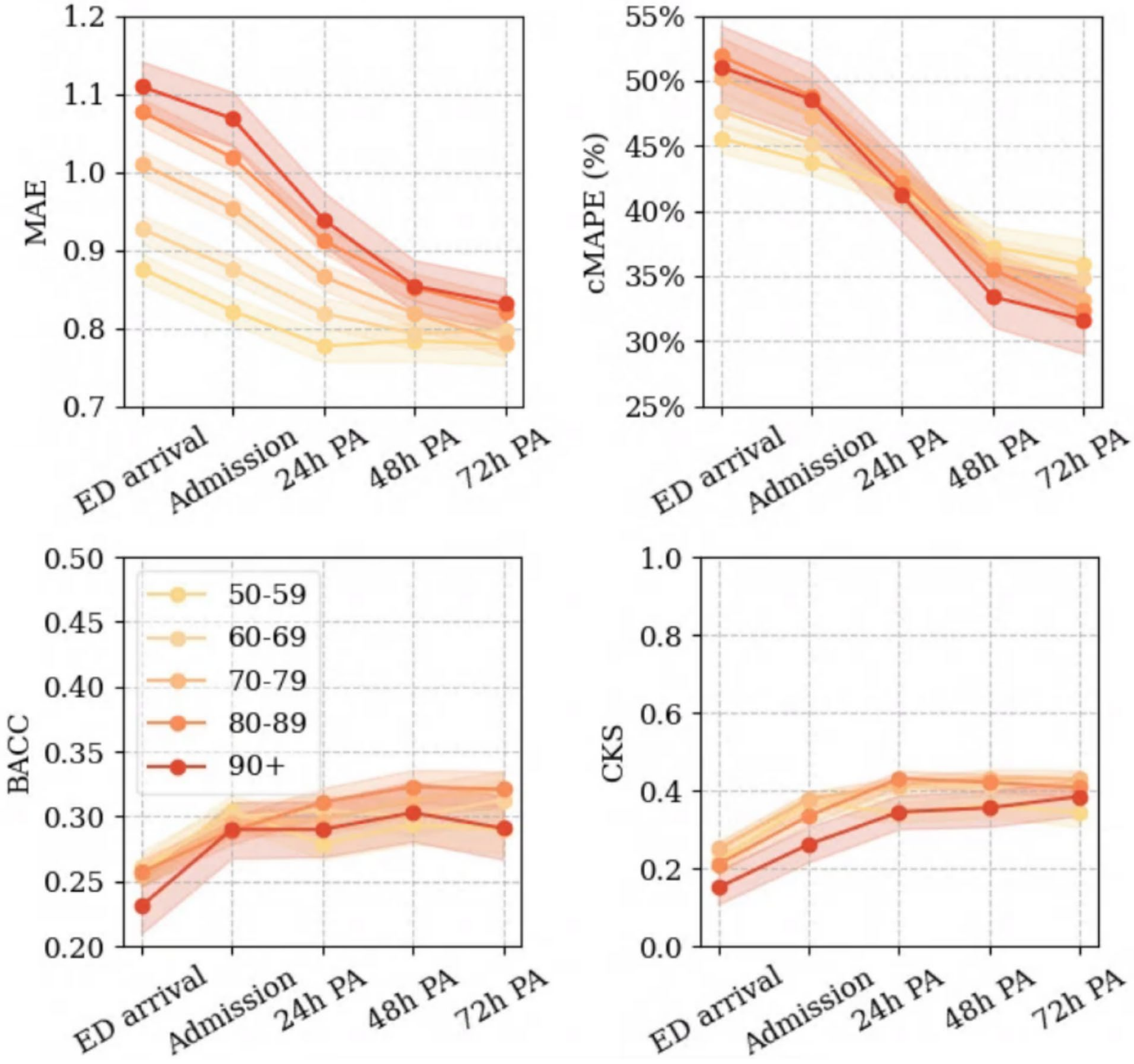

Supplementary Fig. 7. Performance trajectory curves for healthcare contact prediction, stratified by age group. Reported with 95% confidence intervals across the five prediction timepoints. BACC and CKS were estimated after quintile-based discretisation of predictions into five frequency levels: 'Very Low', 'Low', 'Medium', 'Medium-high' and 'High'. MAE – Mean Absolute Error, BACC – Balanced Accuracy, CKS – Cohen's Kappa Score, cMAPE – conditional Mean Absolute Percentage Error, estimated by masking 0 values (no linked contacts) from the estimation.

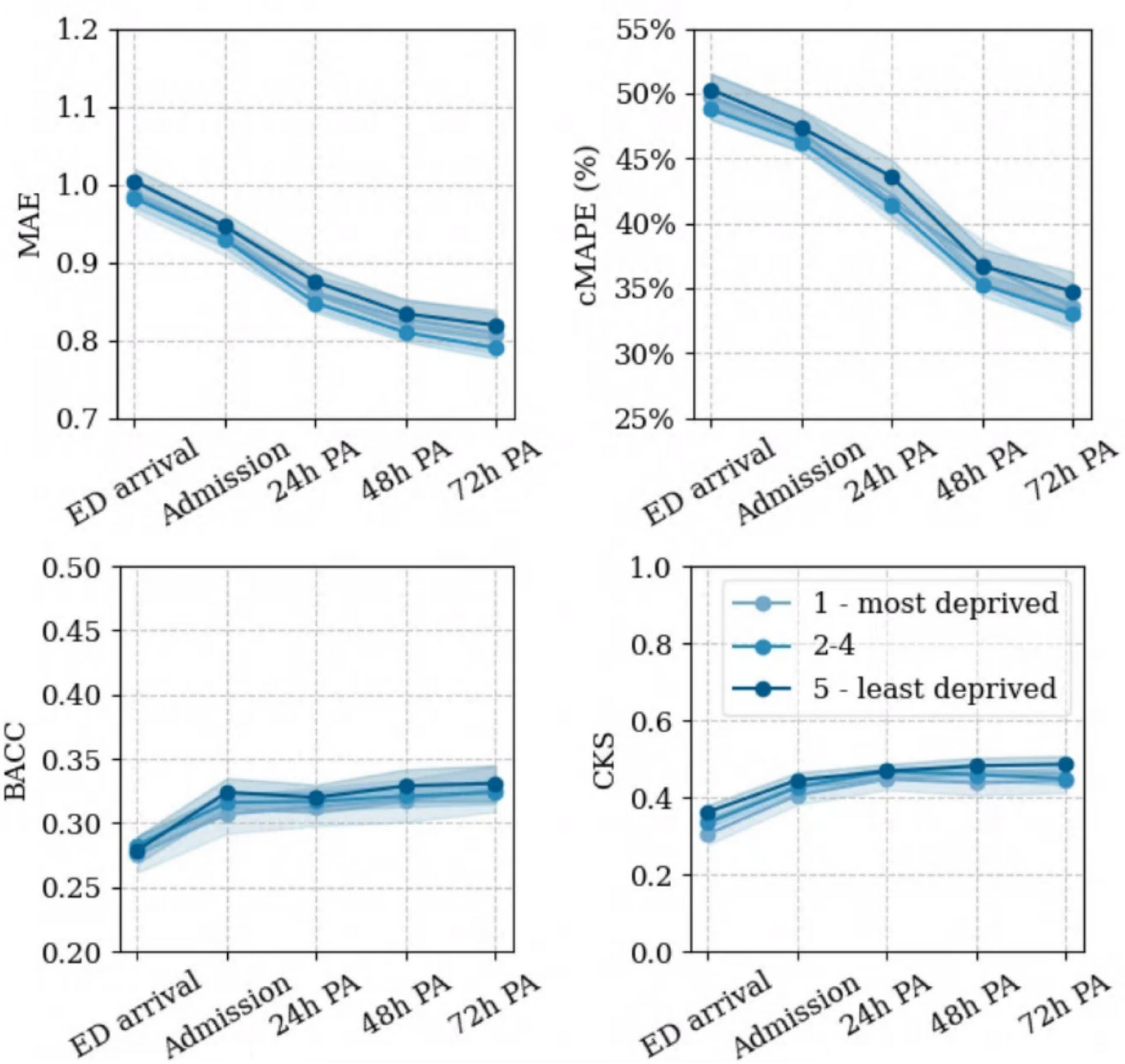

Supplementary Fig. 8. Performance trajectory curves for healthcare contact prediction, stratified by deprivation level. Reported with 95% confidence intervals across the five prediction timepoints. BACC and CKS were estimated after quintile-based discretisation of predictions into five frequency levels: ‘Very Low’, ‘Low’, ‘Medium’, ‘Medium-high’ and ‘High’. MAE – Mean Absolute Error, BACC – Balanced Accuracy, CKS – Cohen’s Kappa Score, cMAPE – conditional Mean Absolute Percentage Error, estimated by masking 0 values (no linked contacts) from the estimation. SIMD – Scottish Index of Multiple Deprivation.

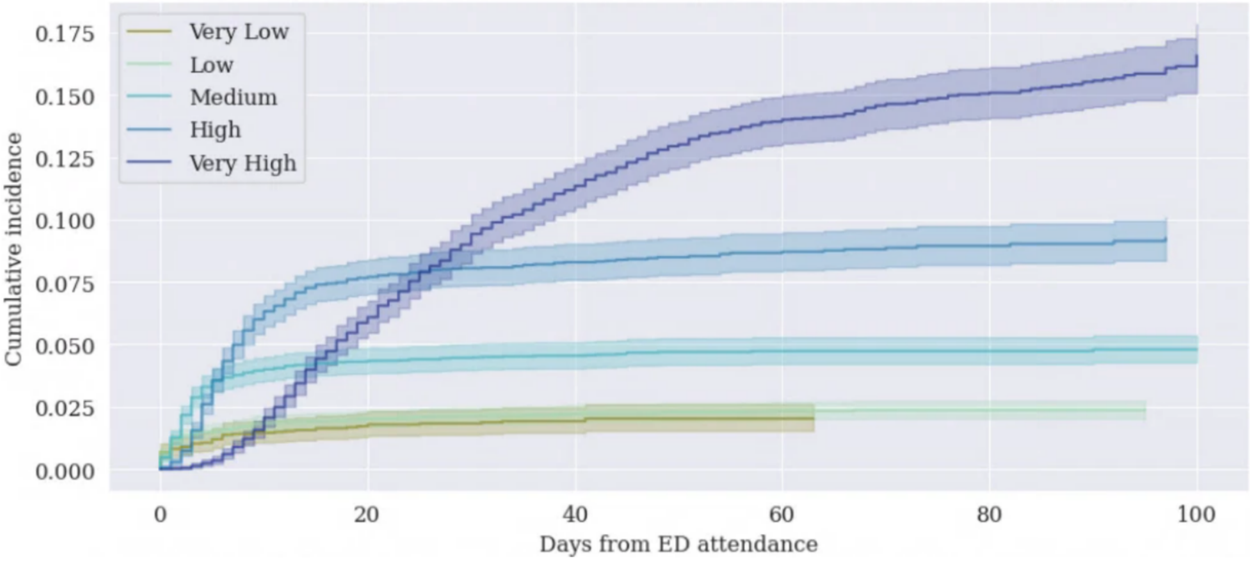

At Risk Per Contact frequency level

Days from ED arrival	Very Low	Low	Medium	High	Very High
0	2,966	8,030	6,520	6,034	5,513
5	1,382	5,527	5,380	2,574	1,028
10	131	296	656	2,407	756
15	53	105	128	627	738
20	33	56	41	168	610
40	4	8	14	21	220
60	1	3	3	5	97
100	0	0	0	1	44

Supplementary Fig. 9. Aalen-Johansen cumulative incidence function of in-hospital death stratified by healthcare contact level, adjusted for non-home discharge outcomes. The table indicates the number of patients at risk of in-hospital death (remaining in the study) over the validation set for the healthcare contact prediction model at point of ED attendance.

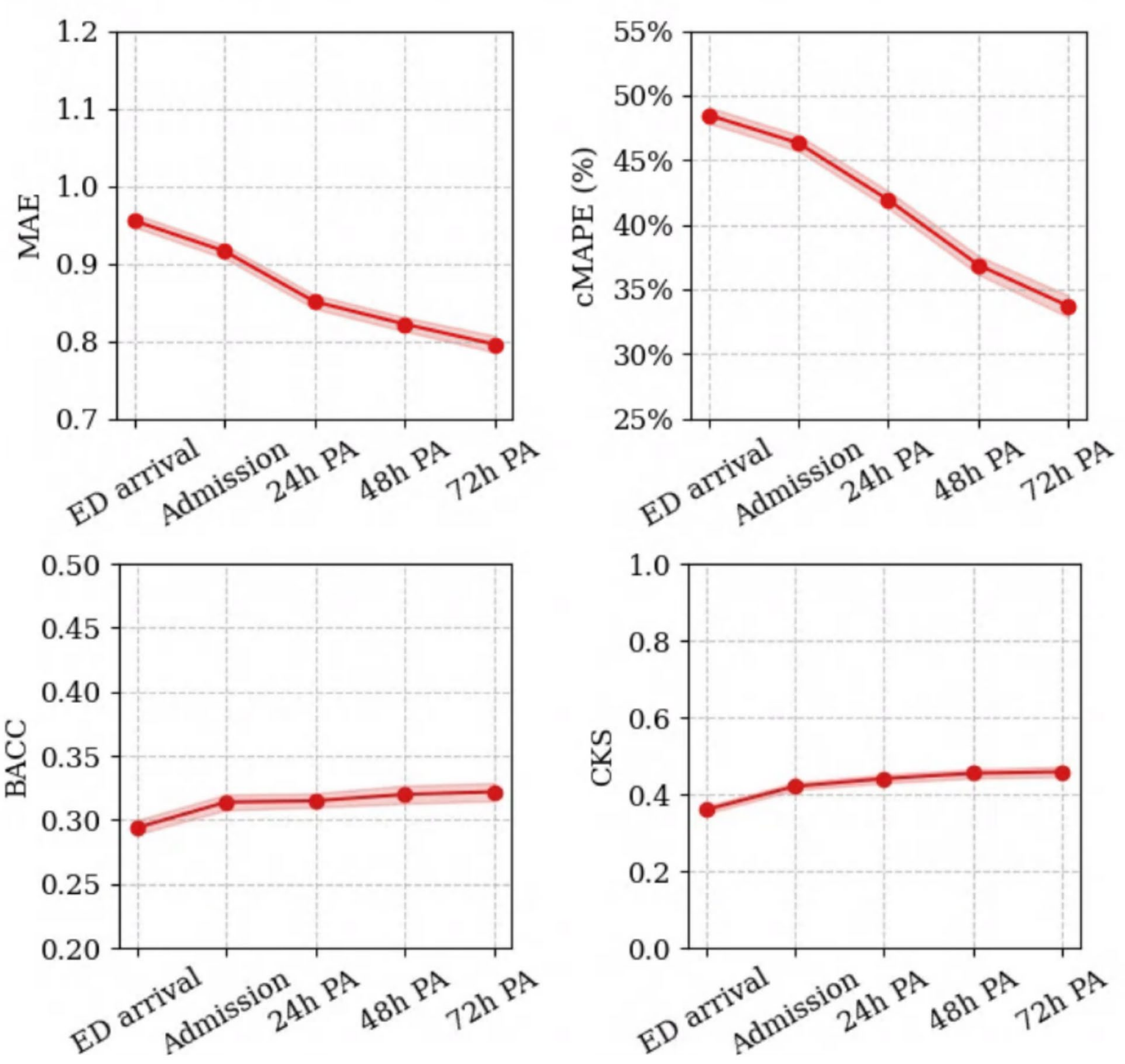

Supplementary Fig. 10. Performance trajectory curves for healthcare contact prediction measured in survivors to discharge. Reported with 95% confidence intervals across the five prediction timepoints. BACC and CKS were estimated after quintile-based discretisation of predictions into five frequency levels: 'Very Low', 'Low', 'Medium', 'Medium-high' and 'High'. MAE – Mean Absolute Error, BACC – Balanced Accuracy, CKS – Cohen's Kappa Score, cMAPE – conditional Mean Absolute Percentage Error, estimated by masking 0 values (no linked contacts) from the estimation.

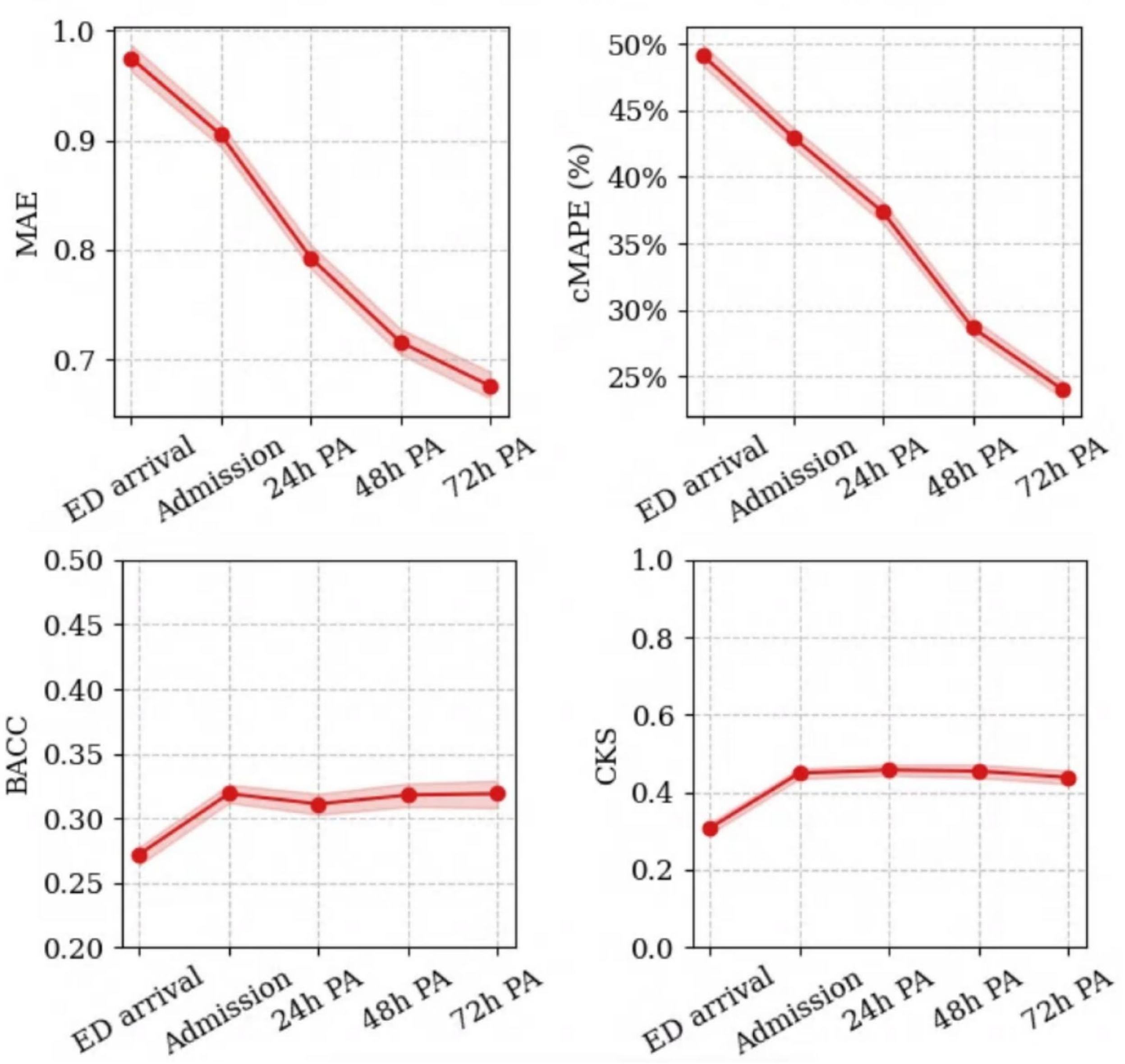

Supplementary Fig. 11. Performance trajectory curves for healthcare contact prediction validated in patients admitted during the COVID-19 lockdown period within the UK (26th Mar 2020 – 19th Jul 2021), using training data on patients admitted before this period. Reported with 95% confidence intervals across the five prediction timepoints. BACC and CKS were estimated after quintile-based discretisation of predictions into five frequency levels: 'Very Low', 'Low', 'Medium', 'Medium-high' and 'High'. MAE – Mean Absolute Error, BACC – Balanced Accuracy, CKS – Cohen's Kappa Score, cMAPE – conditional Mean Absolute Percentage Error, estimated by masking 0 values (no linked contacts) from the estimation. SIMD – Scottish Index of Multiple Deprivation.

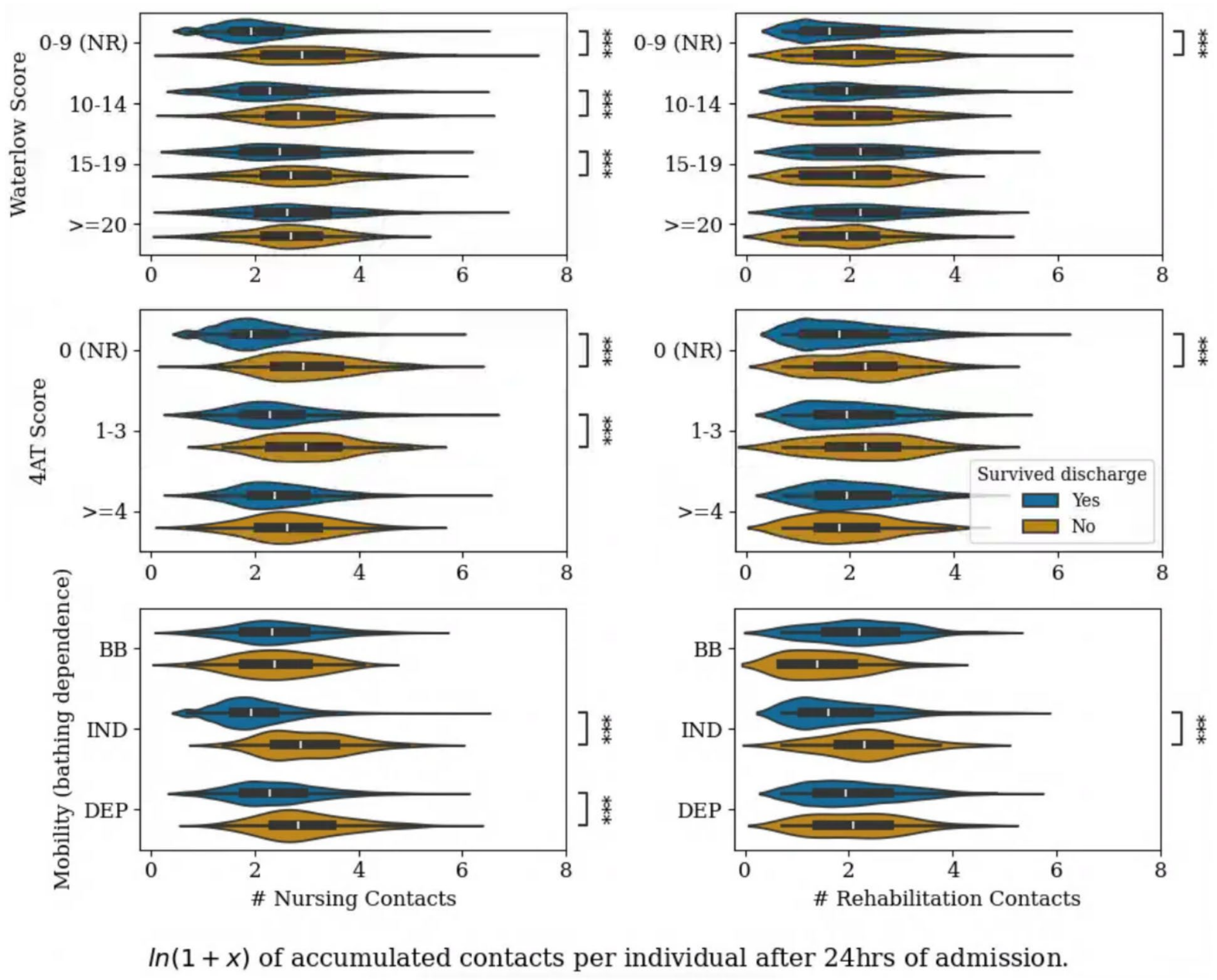

Supplementary Fig. 12. Violin plot showing the distribution of log-transformed nursing and rehabilitation contacts per individual in the validation set, grouped by surviving discharge across the three main predictors within the nursing risk assessments: Waterlow score, 4AT Score and mobility (bathing dependence). Estimates are shown for patients with a documented risk assessment. Statistical testing: Two-sided Mann-Whitney-Wilcoxon test with Bonferroni correction with significance presented at $p<0.001$. Score thresholds – Waterlow: 0-9 (NR) – No Risk, n=10136; 10-14 – Moderate Risk, n=3811; 15-19 – High Risk, n=1429; >=20 – Very High Risk, n=492; 4AT: 0 (NR) – No Risk, n=8286; 1-3 – Possible cognitive impairment, n=1318; >=4 – At Risk of delirium, n=1147; Mobility (bathing dependence): BB – Bed-bound (assessment not applicable), n=258; IND – Independent (no assistance), n=4202; DEP – Dependent (requiring assistance), n=3046.

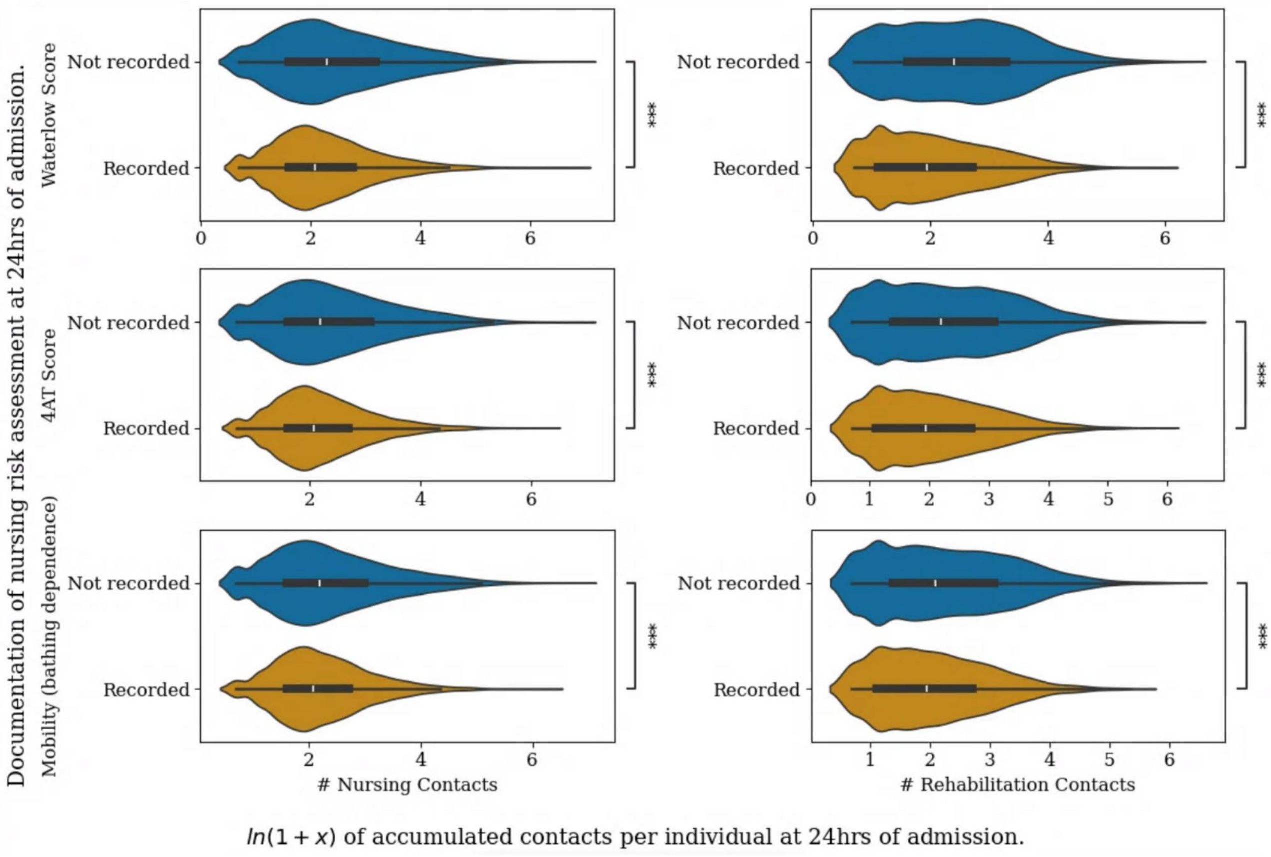

Supplementary Fig. 13. Violin plot showing the distribution of log-transformed nursing and rehabilitation contacts per individual in the validation set, grouped by the documentation of a nursing risk assessment. Statistical testing: Two-sided Mann-Whitney-Wilcoxon test with Bonferroni correction with significance presented at $p<0.001$. Prevalence of recording – Waterlow Score (Not recorded: n=8441, Recorded: n=15868); 4AT Score (Not recorded: n=13565, Recorded: n=10751); Mobility (Not recorded: n=16810, Recorded: n=7506).

49 Spadina Ave. Suite 200
Toronto ON M5V 2J1 Canada
www.biorender.com

Confirmation of Publication and Licensing Rights - Open Access

October 7th, 2025

Subscription Type: *Student Plan - Academic*
Agreement number: *DC28UFJ5TH*
Publisher Name: *NPJ Digital Medicine*

Figure Title: *Figure 1. Flow diagram detailing the feature collection windows and individual prediction timepoints used to forecast healthcare contacts and related hospital outcomes.*

Citation to Use: *Created in BioRender. Georgiev, K. (2025) https://BioRender.com/205crtq*

To whom this may concern,

This document ("Confirmation") hereby confirms that Science Suite Inc. dba BioRender ("BioRender") has granted the following BioRender user: Konstantin Georgiev ("User") a BioRender Academic Publication License in accordance with BioRender's Terms of Service and Academic License Terms ("License Terms") to permit such User to do the following on the condition that all requirements in this Confirmation are met:

1) publish their Completed Graphics created in the BioRender Services containing both User Content and BioRender Content (as both are defined in the License Terms) in publications (journals, textbooks, websites, etc.); and
2) sublicense such Completed Graphics under "open access" publication sublicensing models such as CC-BY 4.0 and more restrictive models, so long as the conditions set forth herein are fully met.

Requirements of User:

1) All Completed Graphics to be published in any publication (journals, textbooks, websites, etc.) must be accompanied by the following citation either as a caption, footnote or reference for each figure that includes a Completed Graphic:
"Created in BioRender. Georgiev, K. (2025) https://BioRender.com/205crtq ".
2) All terms of the License Terms including all Prohibited Uses are fully complied with. E.g. For Academic License Users, no commercial uses (beyond publication in journals, textbooks or websites) are permitted without obtaining or switching to a BioRender Industry Plan.
3) A Reader (defined below) may request that the User allow their figure to be a public template for Readers to view, copy, and modify the figure. It is up to the User to determine what level of access to grant.

Open-Access Journal Readers:

Open-Access journal readers ("Reader") who wish to view and/or re-use a particular Completed Graphic

in an Open-Access journal subject to CC-BY sublicensing may do so by clicking on the URL link in the applicable citation for the subject Completed Graphic.

The re-use/modification options below are available after the Reader requests the User to adapt their figure as a BioRender template and the User has granted such access.

1) View-Only/Free Plan Use: A Reader who wishes to only view the Completed Graphic may do so in the BioRender Services as either a BioRender Free Plan user or simply as a viewer. By becoming a BioRender Free Plan user, the Reader may view, modify and re-use the Completed Graphic as permitted under BioRender's Basic License Terms (e.g. personal use only, no publishing or commercial use permitted).
2) Re-Use/Publish with No Modifications: For any re-use and re-publication of a Completed Graphic with no modification(s) to the Completed Graphic made by the Reader, a Reader may do so by citing the original author using the citation noted above with the Completed Graphic. The Reader must also comply with the underlying License Terms which apply to the Completed Graphic as noted above (e.g. no commercial use for Academic License).
3) Re-Use/Publish with Modifications: For any re-use and re-publication of a Completed Graphic with a modification(s) made by the Reader, the Reader may do so by becoming a BioRender user themselves under either an Academic or Industry Plan, citing the original author using the citation noted above with the Completed Graphic and complying with the applicable License Terms.

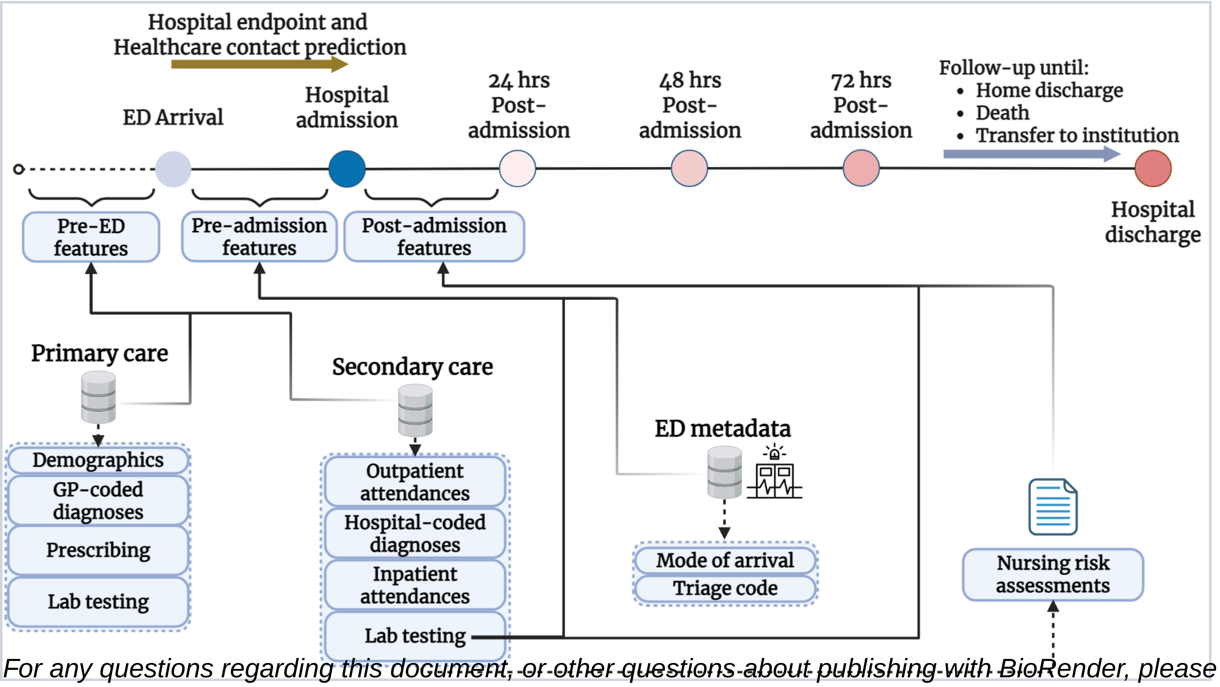

For any questions regarding this document, or other questions about publishing with BioRender, please refer to our BioRender Publication Guide, or contact BioRender Support at support@biorender.com.

49 Spadina Ave. Suite 200
Toronto ON M5V 2J1 Canada
www.biorender.com

Confirmation of Publication and Licensing Rights - Open Access

October 7th, 2025

Subscription Type: *Student Plan - Academic*
Agreement number: *PY28UFIY5H*
Publisher Name: *NPJ Digital Medicine*

Figure Title: *Figure 2. Overview of the data preprocessing, training and evaluation strategy.*

Citation to Use: *Created in BioRender. Georgiev, K. (2025) https://BioRender.com/b80mij6*

To whom this may concern,

This document ("Confirmation") hereby confirms that Science Suite Inc. dba BioRender ("BioRender") has granted the following BioRender user: Konstantin Georgiev ("User") a BioRender Academic Publication License in accordance with BioRender's Terms of Service and Academic License Terms ("License Terms") to permit such User to do the following on the condition that all requirements in this Confirmation are met:

1) publish their Completed Graphics created in the BioRender Services containing both User Content and BioRender Content (as both are defined in the License Terms) in publications (journals, textbooks, websites, etc.); and
2) sublicense such Completed Graphics under "open access" publication sublicensing models such as CC-BY 4.0 and more restrictive models, so long as the conditions set forth herein are fully met.

Requirements of User:

1) All Completed Graphics to be published in any publication (journals, textbooks, websites, etc.) must be accompanied by the following citation either as a caption, footnote or reference for each figure that includes a Completed Graphic:
"Created in BioRender. Georgiev, K. (2025) https://BioRender.com/b80mij6 ".
2) All terms of the License Terms including all Prohibited Uses are fully complied with. E.g. For Academic License Users, no commercial uses (beyond publication in journals, textbooks or websites) are permitted without obtaining or switching to a BioRender Industry Plan.
3) A Reader (defined below) may request that the User allow their figure to be a public template for Readers to view, copy, and modify the figure. It is up to the User to determine what level of access to grant.

Open-Access Journal Readers:

Open-Access journal readers ("Reader") who wish to view and/or re-use a particular Completed Graphic in an Open-Access journal subject to CC-BY sublicensing may do so by clicking on the URL link in the

applicable citation for the subject Completed Graphic.

The re-use/modification options below are available after the Reader requests the User to adapt their figure as a BioRender template and the User has granted such access.

1) View-Only/Free Plan Use: A Reader who wishes to only view the Completed Graphic may do so in the BioRender Services as either a BioRender Free Plan user or simply as a viewer. By becoming a BioRender Free Plan user, the Reader may view, modify and re-use the Completed Graphic as permitted under BioRender's Basic License Terms (e.g. personal use only, no publishing or commercial use permitted).
2) Re-Use/Publish with No Modifications: For any re-use and re-publication of a Completed Graphic with no modification(s) to the Completed Graphic made by the Reader, a Reader may do so by citing the original author using the citation noted above with the Completed Graphic. The Reader must also comply with the underlying License Terms which apply to the Completed Graphic as noted above (e.g. no commercial use for Academic License).
3) Re-Use/Publish with Modifications: For any re-use and re-publication of a Completed Graphic with a modification(s) made by the Reader, the Reader may do so by becoming a BioRender user themselves under either an Academic or Industry Plan, citing the original author using the citation noted above with the Completed Graphic and complying with the applicable License Terms.

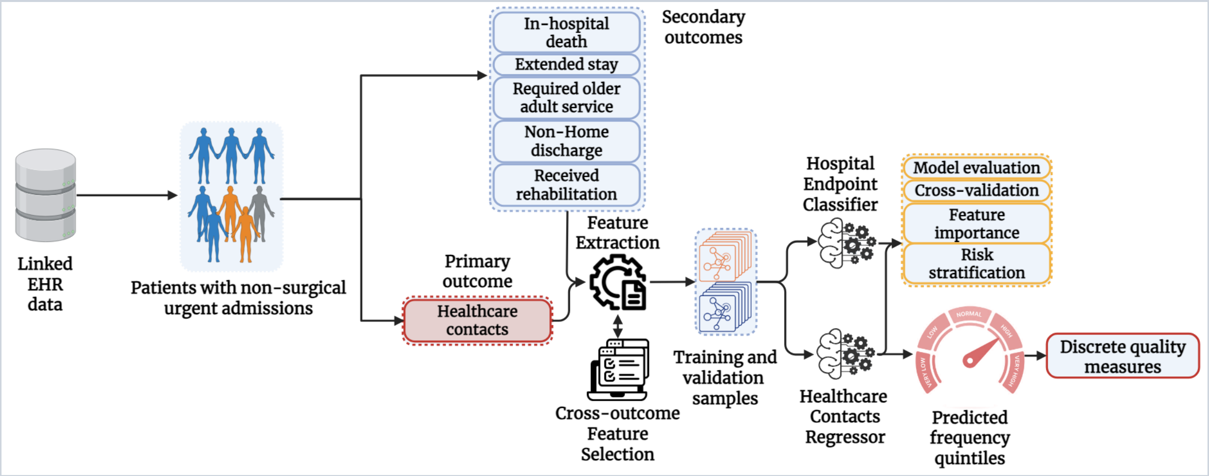

For any questions regarding this document, or other questions about publishing with BioRender, please refer to our BioRender Publication Guide, or contact BioRender Support at support@biorender.com.

Section/Topic	Item	Development / evaluation[1]	Checklist item	Reported on page
**TITLE**				
Title	1	D;E	Identify the study as developing or evaluating the performance of a multivariable prediction model, the target population, and the outcome to be predicted	Title page
**ABSTRACT**				
Abstract	2	D;E	See TRIPOD+AI for Abstracts checklist	Abstract
**INTRODUCTION**				
Background	3a	D;E	Explain the healthcare context (including whether diagnostic or prognostic) and rationale for developing or evaluating the prediction model, including references to existing models	Page 2
	3b	D;E	Describe the target population and the intended purpose of the prediction model in the context of the care pathway, including its intended users (e.g., healthcare professionals, patients, public)	Page 1,2
	3c	D;E	Describe any known health inequalities between sociodemographic groups	Page 2
Objectives	4	D;E	Specify the study objectives, including whether the study describes the development or validation of a prediction model (or both)	Page 2
**METHODS**				
Data	5a	D;E	Describe the sources of data separately for the development and evaluation datasets (e.g., randomised trial, cohort, routine care or registry data), the rationale for using these data, and representativeness of the data	Pages 18-20
	5b	D;E	Specify the dates of the collected participant data, including start and end of participant accrual; and, if applicable, end of follow-up	Page 18
Participants	6a	D;E	Specify key elements of the study setting (e.g., primary care, secondary care, general population) including the number and location of centres	Page 18
	6b	D;E	Describe the eligibility criteria for study participants	Page 18
	6c	D;E	Give details of any treatments received, and how they were handled during model development or evaluation, if relevant	Page 18
Data preparation	7	D;E	Describe any data pre-processing and quality checking, including whether this was similar across relevant sociodemographic groups	Pages 21,22
Outcome	8a	D;E	Clearly define the outcome that is being predicted and the time horizon, including how and when assessed, the rationale for choosing this outcome, and whether the method of outcome assessment is consistent across sociodemographic groups	Page 21
	8b	D;E	If outcome assessment requires subjective interpretation, describe the qualifications and demographic characteristics of the outcome assessors	Not applicable
	8c	D;E	Report any actions to blind assessment of the outcome to be predicted	Not applicable
Predictors	9a	D	Describe the choice of initial predictors (e.g., literature, previous models, all available predictors) and any pre-selection of predictors before model building	Page 19-20, Supplementary Table 1
	9b	D;E	Clearly define all predictors, including how and when they were measured (and any actions to blind assessment of predictors for the outcome and other predictors)	Supplementary Table 1
	9c	D;E	If predictor measurement requires subjective interpretation, describe the qualifications and demographic characteristics of the predictor assessors	Not applicable
Sample size	10	D;E	Explain how the study size was arrived at (separately for development and evaluation), and justify that the study size was sufficient to answer the research question. Include details of any sample size calculation	Page 18
Missing data	11	D;E	Describe how missing data were handled. Provide reasons for omitting any data	Page 22
Analytical methods	12a	D	Describe how the data were used (e.g., for development and evaluation of model performance) in the analysis, including whether the data were partitioned, considering any sample size requirements	Page 26
	12b	D	Depending on the type of model, describe how predictors were handled in the analyses (functional form, rescaling, transformation, or any standardisation).	Page 22, 24
	12c	D	Specify the type of model, rationale[2], all model-building steps, including any hyperparameter tuning, and method for internal validation	Pages 23-25
	12d	D;E	Describe if and how any heterogeneity in estimates of model parameter values and model performance was handled and quantified across clusters (e.g., hospitals, countries). See TRIPOD-Cluster for additional considerations[3]	Not applicable
	12e	D;E	Specify all measures and plots used (and their rationale) to evaluate model performance (e.g., discrimination, calibration, clinical utility) and, if relevant, to compare multiple models	Pages 23-25
	12f	E	Describe any model updating (e.g., recalibration) arising from the model evaluation, either overall or for particular sociodemographic groups or settings	Page 24
	12g	E	For model evaluation, describe how the model predictions were calculated (e.g., formula, code, object, application programming interface)	Page 24,25
Class imbalance	13	D;E	If class imbalance methods were used, state why and how this was done, and any subsequent methods to recalibrate the model or the model predictions	Page 26
Fairness	14	D;E	Describe any approaches that were used to address model fairness and their rationale	Page 24
Model output	15	D	Specify the output of the prediction model (e.g., probabilities, classification). Provide details and rationale for any classification and how the thresholds were identified	Page 24

[1] D=items relevant only to the development of a prediction model; E=items relating solely to the evaluation of a prediction model; D;E=items applicable to both the development and evaluation of a prediction model

[2] Separately for all model building approaches.

[3] TRIPOD-Cluster is a checklist of reporting recommendations for studies developing or validating models that explicitly account for clustering or explore heterogeneity in model performance (eg, at different hospitals or centres). Debray et al, BMJ 2023; 380: e071018 [DOI: 10.1136/bmj-2022-071018]

Training versus evaluation	16	D;E	Identify any differences between the development and evaluation data in healthcare setting, eligibility criteria, outcome, and predictors	Page 5; Supplementary Table 9
Ethical approval	17	D;E	Name the institutional research board or ethics committee that approved the study and describe the participant-informed consent or the ethics committee waiver of informed consent	Page 27
**OPEN SCIENCE**				
Funding	18a	D;E	Give the source of funding and the role of the funders for the present study	Page 28
Conflicts of interest	18b	D;E	Declare any conflicts of interest and financial disclosures for all authors	Page 29
Protocol	18c	D;E	Indicate where the study protocol can be accessed or state that a protocol was not prepared	Not applicable
Registration	18d	D;E	Provide registration information for the study, including register name and registration number, or state that the study was not registered	Not applicable
Data sharing	18e	D;E	Provide details of the availability of the study data	Page 28
Code sharing	18f	D;E	Provide details of the availability of the analytical code[4]	Page 28
**PATIENT & PUBLIC INVOLVEMENT**				
Patient & Public Involvement	19	D;E	Provide details of any patient and public involvement during the design, conduct, reporting, interpretation, or dissemination of the study or state no involvement.	Not applicable
**RESULTS**				
Participants	20a	D;E	Describe the flow of participants through the study, including the number of participants with and without the outcome and, if applicable, a summary of the follow-up time. A diagram may be helpful.	Figure 1,2
	20b	D;E	Report the characteristics overall and, where applicable, for each data source or setting, including the key dates, key predictors (including demographics), treatments received, sample size, number of outcome events, follow-up time, and amount of missing data. A table may be helpful. Report any differences across key demographic groups.	Page 3,4 Table 1
	20c	E	For model evaluation, show a comparison with the development data of the distribution of important predictors (demographics, predictors, and outcome).	Supplementary Table 9
Model development	21	D;E	Specify the number of participants and outcome events in each analysis (e.g., for model development, hyperparameter tuning, model evaluation)	Supplementary Table 3-7,9
Model specification	22	D	Provide details of the full prediction model (e.g., formula, code, object, application programming interface) to allow predictions in new individuals and to enable third-party evaluation and implementation, including any restrictions to access or re-use (e.g., freely available, proprietary)[5]	Supplementary Table 8-11
Model performance	23a	D;E	Report model performance estimates with confidence intervals, including for any key subgroups (e.g., sociodemographic). Consider plots to aid presentation.	Pages 5-8, Table 3, Figure 3,4
	23b	D;E	If examined, report results of any heterogeneity in model performance across clusters. See TRIPOD Cluster for additional details[3].	Not applicable
Model updating	24	E	Report the results from any model updating, including the updated model and subsequent performance	Supplementary Figire 8-11
**DISCUSSION**				
Interpretation	25	D;E	Give an overall interpretation of the main results, including issues of fairness in the context of the objectives and previous studies	Page 10
Limitations	26	D;E	Discuss any limitations of the study (such as a non-representative sample, sample size, overfitting, missing data) and their effects on any biases, statistical uncertainty, and generalizability	Page 14-16
Usability of the model in the context of current care	27a	D	Describe how poor quality or unavailable input data (e.g., predictor values) should be assessed and handled when implementing the prediction model	Page 15
	27b	D	Specify whether users will be required to interact in the handling of the input data or use of the model, and what level of expertise is required of users	Page 14
	27c	D;E	Discuss any next steps for future research, with a specific view to applicability and generalizability of the model	Pages 15-17

From: Collins GS, Moons KGM, Dhiman P, et al. *BMJ* 2024;385:e078378. doi:10.1136/bmj-2023-078378

[4] This relates to the analysis code, for example, any data cleaning, feature engineering, model building, evaluation.
[5] This relates to the code to implement the model to get estimates of risk for a new individual.
